# Carbon sources and pathways for citrate secreted by human prostate cancer cells determined by NMR tracing and metabolic modeling

**DOI:** 10.1073/pnas.2024357119

**Published:** 2022-03-30

**Authors:** Frits H. A. van Heijster, Vincent Breukels, Kees (C.) F. J. Jansen, Jack A. Schalken, Arend Heerschap

**Affiliations:** ^a^Department of Radiology and Nuclear Medicine, Radboud University Medical Center, 6525 GA Nijmegen, The Netherlands;; ^b^Department of Urology, Radboud University Medical Center, 6525 GA Nijmegen, The Netherlands

**Keywords:** prostate, citrate, carbon-13, Krebs cycle, cancer

## Abstract

Prostate epithelial cells have the unique capacity to secrete large amounts of citrate, but the carbon sources and metabolic pathways that maintain this production are not well known. We mapped potential pathways for citrate carbons in the human prostate cancer metastasis cell lines LNCaP and VCaP, for which we first established that they secrete citrate (For LNCaP 5.6 ± 0.9 nmol/h per 10^6^ cells). Using ^13^C-labeled substrates, we traced the incorporation of ^13^C into citrate by NMR of extracellular fluid. Our results provide direct evidence that glucose is a main carbon source for secreted citrate. We also demonstrate that carbons from supplied glutamine flow via oxidative Krebs cycle and reductive carboxylation routes to positions in secreted citrate but likely do not contribute to its net synthesis. The potential anaplerotic carbon sources aspartate and asparagine did not contribute to citrate carbons. We developed a quantitative metabolic model employing the ^13^C distribution in extracellular citrate after ^13^C glucose and pyruvate application to assess intracellular pathways of carbons for secreted citrate. From this model, it was estimated that in LNCaP about 21% of pyruvate entering the Krebs cycle is converted via pyruvate carboxylase as an anaplerotic route at a rate more than sufficient to compensate carbon loss of this cycle by citrate secretion. This model provides an estimation of the fraction of molecules, including citrate, leaving the Krebs cycle at every turn. The measured ratios of ^13^C atoms at different positions in extracellular citrate may serve as biomarkers for (malignant) epithelial cell metabolism.

## Significance

The human prostate accumulates high luminal citrate levels to serve sperm viability. There is only indirect qualitative evidence about metabolic pathways and carbon sources maintaining these levels. Human citrate-secreting prostate cancer cells were supplied with ^13^C-labeled substrates, and NMR spectra of extracellular fluid were recorded. We report absolute citrate production rates of prostate cells and direct evidence that glucose is the main carbon source for secreted citrate. Pyruvate carboxylase provides sufficient anaplerotic carbons to support citrate secretion. Glutamine carbons exchange with carbons for secreted citrate but are likely not involved in its net synthesis. Moreover, we developed metabolic models employing the ^13^C distribution in extracellular citrate as input to assess intracellular pathways followed by carbons toward citrate.

**Fig. 1.**
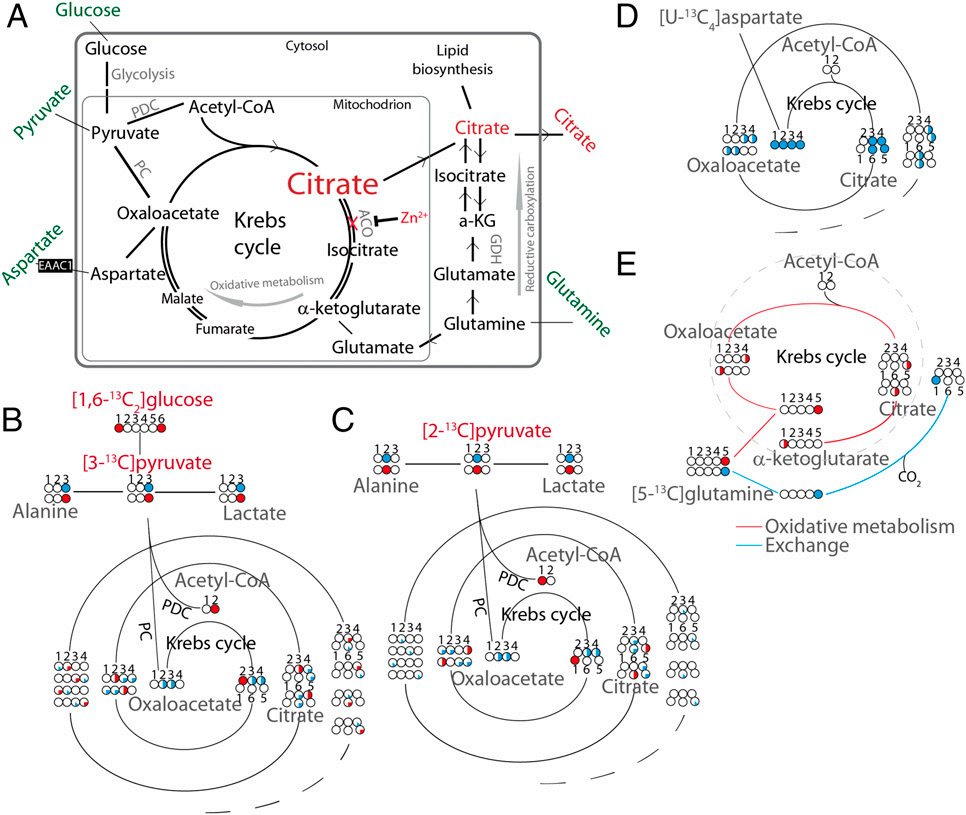
Schematic overview of metabolic pathways relevant in the biosynthesis of citrate in the prostate and schematic representation of ^13^C atoms in compounds entering the Krebs cycle and their fate during multiple cycle turns to produce citrate. (*A*) In healthy prostate epithelial cells, zinc ions, imported via the ZIP1 transporter, inhibit aconitase, the enzyme catalyzing the stereospecific isomerization of citrate into isocitrate. The resulting excess of citrate is transported to the cytosol via the citrate transporter (CT) and excreted by the cells via the citrate transporter KCitT into the luminal space. Upon malignant transformation, Zn^2+^ uptake is decreased and consequently inhibition of aconitase is released and citrate becomes more oxidized to isocitrate instead of being secreted. Potential metabolites supplying the Krebs cycle with carbons are indicated in green. Anaplerosis is needed when citrate secretion drains carbons from this cycle. Aspartate can enter the Krebs cycle via oxaloacetate. Glutamine is converted into glutamate and its carbons enter the Krebs cycle as five-carbon metabolite α-ketoglutarate. Pyruvate can be converted by PC into four-carbon Krebs cycle intermediate oxaloacetate. Both reductive and oxidative metabolic pathways are indicated. (*B*) The distribution of ^13^C labels originating from [1,6-^13^C_2_]glucose. (*C*) Idem from [2-^13^C]pyruvate. Pyruvate carbons converted to oxaloacetate by PC are indicated by blue circles and those converted into acetyl-CoA by PDC by red circles. (*D*) Distribution of ^13^C labels in possible anaplerosis involving [U-^13^C_4_]aspartate. (*E*) Idem involving [5-^13^C]glutamine. Glutamine carbons converted by oxidative metabolism are indicated by red circles and those converted by reductive exchange by blue circles. In this figure the carbon numbers in each compound are indicated and for simplicity mitochondrial and cellular export of citrate is omitted. Citrate carbon numbering is chosen in such a way that C1 and C2 of acetyl-CoA end up at C1 and C2 of citrate.

Healthy prostate epithelial cells have the unique capability of secreting citrate into the ducts of the prostate (1). Citrate is formed by condensation of oxaloacetate and acetyl-CoA, catalyzed by citrate synthase as the first step of the Krebs cycle. A part of this citrate is diverted from the Krebs cycle into the cytosol, facilitated by a citrate transport protein in the inner mitochondrial membrane. It can then be secreted into the ducts of the prostate, involving an independent electrogenic transport system (2). Citrate in the lumen of the prostate can reach levels up to about 180 mM, which is thought to mainly serve as an energy source for sperm cells (3, 4). Citrate accumulation is promoted by inhibition of the citrate-converting enzyme m-aconitase through binding of zinc, which is taken up at relatively high levels in epithelial prostate cells (1, 5, 6) (Fig. 1*A*).

Prostate cancer is a major health burden worldwide (7). A remarkable metabolic change upon malignant transformation in the prostate is the local decrease of the tissue concentration of citrate. This can be a result of tumor cells occupying space in ducts, physically displacing luminal fluid, and/or of metabolic reprogramming toward the diversion of less citrate (8). A key metabolic event in the transformation of epithelial cells to cancer cells is considered to be down-regulation of the zinc transporter ZIP1, causing decreased zinc uptake and therefore increased m-aconitase activity and oxidation of citrate instead of secretion into ducts (Fig. 1*A*) (9). A decreased citrate signal in MR spectroscopic images of the prostate is employed as biomarker to identify cancer lesions (10–13). Understanding metabolic reprogramming in malignant transformation may help to better diagnose and treat prostate cancer (14–18).

A major question is how a high level of citrate production is supported by the metabolic network of the prostate. Studies in epithelial cells and ventral tissue of the rat prostate point to glucose as a major carbon source for citrate (19, 20). Rat epithelial prostate cells only produced citrate with aspartate in the medium, suggesting that aspartate is an essential precursor (21). Its uptake is mediated by the amino acid transporter EAAC1, and subsequent transamination by mitochondrial aspartate aminotransferase (mAAT) provides oxaloacetate (22). The high EAAC1 expression in rat and human prostate further seems to support the importance of aspartate (23). None of these studies, however, provide direct evidence that carbon atoms from glucose or aspartate indeed end up in extracellular citrate and which metabolic pathways are followed by these carbons.

Because citrate produced in the Krebs cycle flows to the luminal space and toward de novo lipogenesis (24), this cycle is depleted of carbon atoms needed for its maintenance and therefore requires anaplerotic supplementation. In the case that glucose is a major carbon source for citrate, aspartate may function as an anaplerotic supply. Anaplerosis can also occur via pyruvate and glutamine (25) (Fig. 1*A*). Pyruvate can be converted into oxaloacetate by pyruvate carboxylase (PC) and glutamine into glutamate by glutaminase and subsequently into the Krebs cycle intermediate α-ketoglutarate by glutamate dehydrogenase (GDH). Since prostate cells harbor a high number of glutamine transporters (26) and glutamine occurs at relatively high levels in blood, it may serve as carbon source for citrate.

To determine which of the abovementioned substrates and metabolic pathways may contribute carbons in the production of citrate in the human prostate we searched for prostate epithelial cells of human origin that are able to secrete citrate in sufficient amounts for metabolic analysis. Previous studies indicated that the prostate cancer lymph node metastatic cell line LNCaP secretes citrate (27, 28). LNCaP cells also express the amino acid transporter EAAC1 and are commonly used as a model to study prostate cancer metabolism (23). VCaP is a prostate cancer cell line derived from a vertebral bone metastasis (29). Like LNCaP cells, the VCaP cells express the glutamine transporter ASCT2 (30, 31), are androgen-sensitive, and produce prostate-specific antigen (PSA), reflecting their prostatic origin and still-differentiated nature (32–34), and thus may also secrete citrate.

An elegant method to follow metabolism in tissues and cells is by supplying them with ^13^C-labeled substrates and to monitor the fate of the ^13^C atoms by ^13^C NMR spectroscopy (35–37). The specific ^13^C labeling of metabolic products provides information on their synthetic route. In this study we focused on the specific ^13^C labeling pattern of secreted citrate as a readout of intracellular metabolic pathways contributing to its carbon skeleton. Eventually, the labeling pattern of extracellular citrate may be used as a fingerprint of intracellular metabolism for diagnostic purposes.

After establishing that LNCaP and VCaP cells indeed secrete citrate, we investigated whether and how aspartate, asparagine, glucose, pyruvate, and glutamine can serve as carbon sources for this extracellular citrate. For this purpose, we employed one-dimensional (1D) and two-dimensional (2D) ^13^C and ^1^H NMR of the growth media of the VCaP and LNCaP cells. We developed a metabolic model that uses specific ^13^C labeling of extracellular citrate, after ^13^C glucose and pyruvate supplementation, as input to provide quantitative information of intracellular metabolism supporting supply of citrate carbons. Ultimately, compounds and metabolic pathways involved in citrate production could act as biomarkers to characterize (malignant) epithelial cell metabolism and may be considered as targets for treatments (17, 38, 39).

## Results

**Fig. 2.**
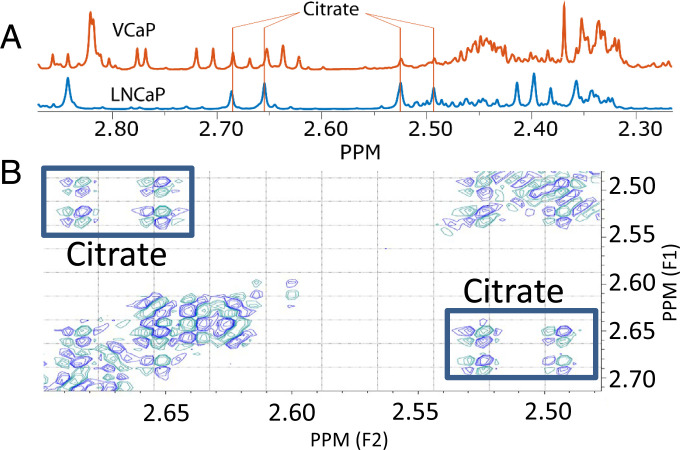
Citrate resonances in ^1^H NMR spectra of cell media. (*A*) Spectra of RPMI-1640 media in which cells grew for 48 h (VCaP: orange; LNCaP: blue). In both spectra the quartet signal of citrate protons is indicated. (*B*) Confirmation of the assignment of citrate resonances by a DQF-COSY spectrum of growth medium from VCaP cells. The cross-peaks of the citrate doublet of doublets are clearly seen at 2.71, 2.68, 2.57, and 2.54 ppm.

All experiments were performed on cells grown to full confluency, before incubation with supplementations for 48 h. Starting with similar numbers of cells, LNCaP reached full confluency about twice as fast as VCaP (about 2 wk vs. 1 mo in 75-cm^2^ flasks). All ^1^H NMR spectra of the cell incubation media of LNCaP and VCaP cells showed the quartet multiplet originating from citrate protons (Fig. 2*A*). The correct assignment of these citrate signals was verified by spiking the sample with citrate and by double-quantum filtered correlated spectroscopy (DQF-COSY) experiments (Fig. 2*B*), showing a doublet of doublets signal, as expected for citrate.

### Citrate Production and Effect of Zn^2+^.

Citrate production by LNCaP was 10 times higher than by VCaP: 5.6 ± 0.95 nmol/h per 10^6^ cells versus 0.43 ± 0.16 nmol/h per 10^6^ cells averaged over 48 h (*SI Appendix*, Table S1).To test if Zn^2+^ in the incubation medium influences citrate secretion of these cells we determined the average production of citrate, per 10^6^ cells over 48 h, and, as additional metabolic sensors, of lactate and alanine (*SI Appendix*, Fig. S1 and Table S1). The presence of 50 µM Zn^2+^ did not change the cell count for either cell line, but LNCaP produced significantly lower amounts of citrate, alanine (*P* < 0.05), and lactate (*P* < 0.01). However, Zn^2+^ supplied to VCaP cells did not affect citrate and alanine production and only slightly increased lactate (*P* < 0.05). The metabolite ratios citrate/lactate and citrate/alanine did not change upon Zn^2+^ addition, but alanine/lactate for LNCaP was significantly increased (*SI Appendix*, Table S1).

### Is Citrate Labeled by Supplementation with [U-^13^C_4_]Aspartate or Asparagine?

**Fig. 3.**
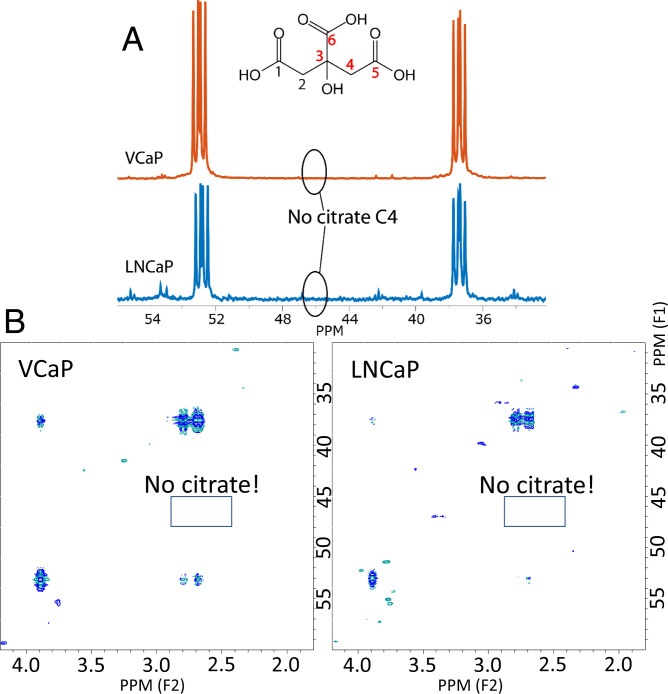
Absence of ^13^C signals for citrate after supplementation with ^13^C aspartate. (*A*) ^13^C NMR spectra and (*B*) ^1^H-^13^C HSQC spectra of RPMI-1640 medium in which VCaP cells or LNCaP cells have been grown for 48 h after supplementation of the medium with [U-^13^C]aspartate (dominant signals in the spectrum). Only the chemical shift range is shown where the ^13^C2/4 signal of citrate is expected. No citrate signal is observed in this spectral range, but signals for citrate are observed in ^1^H NMR spectra (*SI Appendix*, Fig. S6).

To test if aspartate or, alternatively, asparagine are carbon sources for citrate, we provided these compounds, uniformly ^13^C-labeled, to both cell lines in the presence of 11 mM nonlabeled glucose. The aspartate experiments were also performed by replacing glucose with 7 mM nonlabeled pyruvate. The ^13^C labels of aspartate or asparagine may end up in citrate via oxaloacetate and subsequent condensation with acetyl-CoA (Fig. 1*D*). None of these experiments resulted in any detectable ^13^C resonance for citrate in ^13^C-NMR spectra and in 2D ^1^H-^13^C heteronuclear single-quantum coherence (HSQC) spectra of the medium (Fig. 3). However, unlabeled citrate was detected in ^1^H NMR spectra of the same samples, confirming that citrate was produced (*SI Appendix*, Fig. S2). ^13^C NMR spectra of extracts of LNCaP cells supplemented with [^13^C]aspartate did not show detectable levels of labeled aspartate.

### Is Citrate Labeled by Supplementation with [1,6-^13^C_2_]Glucose?

**Fig. 4.**
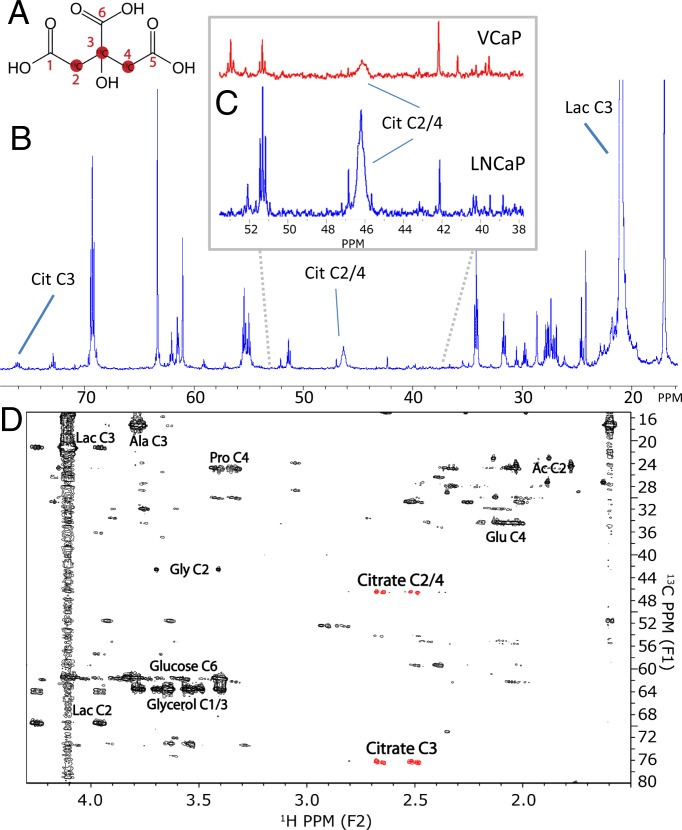
^13^C labeling of citrate after [1,6-^13^C_2_]glucose supplementation and 2D heteronuclear correlation experiment to validate ^13^C citrate signal assignment and to identify ^13^C labeling in other compounds after [1,6-^13^C]glucose supplementation of LNCaP cells. (*A*) Citrate with carbon numbers indicated. (*B*) ^13^C NMR spectra of RPMI-1640 medium of LNCaP cells, after 48 h of supplementation with [1,6-^13^C_2_]glucose. Chemical shift positions of the C2/4 and C3 peaks of citrate and C3 of lactate are indicated. (*C*) Part of ^13^C NMR spectra of medium in which VCaP cells (red) or LNCaP cells (blue) have been grown. In both spectra a broad resonance is observed at ∼46.5 ppm corresponding to ^13^C carbons at the C2 and C4 position of citrate. Both spectra are scaled and referenced to the ^13^C TSP signal at −2.0 ppm. (*D*) ^1^H-^13^C HMBC spectrum of medium showing cross-peaks for protons two or more bonds away from ^13^C (^2^J_CH_, ^3^J_CH_) at the corresponding ^13^C-chemical shifts. Citrate cross-peaks are indicated in red. High intensity is also found for signals of glucose, glycerol, glutamate (Glu), proline (Pro), alanine (Ala), lactate (Lac), acetate (Ac), and glycine (Gly). See also *SI Appendix*, Table S5.

A major potential source of carbons for citrate secreted by prostate cells is glucose. To test if glucose carbons indeed end up in secreted citrate we supplied prostate cells with 11 mM [1,6-^13^C]glucose, of which the ^13^C carbons were expected to label citrate via the Krebs cycle (Fig. 1*B*). In contrast to the experiments with ^13^C aspartate or asparagine as precursor, ^13^C NMR spectra of the medium of LNCaP and VCaP cells supplemented with [1,6-^13^C]glucose for 48 h showed resonances for ^13^C-labeled citrate, in particular at the positions of carbons 2/4 at ∼46.4 ppm and 3 at ∼76.2 ppm (Fig. 4). These resonances were fitted, including the ^13^C–^13^C couplings, to estimate the relative ^13^C integrals of the different carbon atoms of citrate (*SI Appendix*, Fig. S3).

The assignment of these resonances was confirmed by the presence of cross peaks at citrate ^1^H and ^13^C chemical shifts in ^1^H-^13^C HSQC and ^1^H-^13^C HMBC (heteronuclear multiple bond correlation) heteronuclear correlation studies of the medium of LNCaP, supplemented with [1,6-^13^C]glucose (Fig. 4*D*).

### Other ^13^C-Labeled Metabolites Present in the Medium and ^13^C Enrichment of the Pyruvate and Acetyl-CoA Pool after [1,6-^13^C_2_]Glucose Supplementation.

Along with the ^13^C signals for labeled citrate and [1,6-^13^C_2_]glucose itself, the 2D ^1^H-^13^C HSQC and ^1^H-^13^C HMBC heteronuclear correlation spectra of the medium also revealed relatively high ^13^C signal intensities for [3-^13^C]lactate, [3-^13^C]alanine, [4-^13^C]proline, [2-^13^C]glycine, [1/3-^13^C]glycerol, and [4-^13^C]glutamate (Fig. 4*D* and *SI Appendix*, Table S5). Apparently, glucose carbons served for the synthesis of several metabolites secreted by the prostate epithelial cell lines.

The ^13^C enrichment of the pyruvate pool, after supplying the cells with 99% enriched [1,6-^13^C_2_]glucose, was determined from the ^13^C enrichment of lactate, assuming that this reflects pyruvate enrichment since the rate of pyruvate-to-lactate conversion is high. The fractional enrichment of lactate C3 was estimated from ^1^H spectra of the incubation media; the methyl protons of unlabeled lactate appear as a doublet at 1.33 ppm, due to ^3^J_HH_ coupling with the neighboring proton, and those of [^13^C_3_]lactate as a doublet of doublets due to additional ^1^J_CH_ coupling. The [^13^C_3_]lactate-to-[^12^C_3_]lactate ratio for the incubation medium and cell extracts of LNCaP indicated that ∼80% of the pyruvate pool in the cells was ^13^C-labeled. For VCaP, around 75% of the pyruvate pool was ^13^C-labeled (*SI Appendix*, Fig. S4 and Table S2). The ^13^C enrichment of acetyl-CoA, as estimated from the ^13^C_3_ labeling pattern of secreted glutamate, was estimated to be 56% for LNCaP and 50% for VCaP (*SI Appendix*, Table S2).

### Glucose Consumption Rate and Its Implications.

To determine if overall energy metabolism differs between VCaP and LNCaP, their glucose consumption rates were estimated by measuring the depletion of [1,6-^13^C_2_]glucose in incubation media of both cell lines after 48 h. This was done by first integrating the [1,6-^13^C_2_]glucose peaks in the ^13^C NMR spectrum and comparing the integral to the [^13^C]lactate peak integral. Subsequently, the [^13^C]lactate concentration was determined by integrating the ^13^C-labeled lactate peaks in the ^1^H NMR spectrum and comparing that to the integral of the trimethylsilyl propionic acid (TSP) peak (concentration 0.2 mM), taking the number of protons of lactate and TSP into account. About 2.5 times as much glucose was used by LNCaP cells (255 ± 19 nmol/h per 10^6^ cells) as by VCaP cells (93 ± 22 nmol/h per 10^6^ cells) (*SI Appendix*, Table S2).

It is of interest to compare this with the production rate of citrate and other molecules (*SI Appendix*, Fig. S1). As described in *SI Appendix* it is possible to derive from these numbers an estimated oxidative triose consumption rate of ∼250 nmol/h per 10^6^ cells. If 21% proceeds via the PC route (discussed below) this would amount to about 53 nmol/h per 10^6^ cells, which is much larger than the citrate production rate of 1∼10 nmol/h per 10^6^ cells in LNCaP. Hence PC anaplerosis can completely cover carbon loss by citrate secretion. Moreover, the estimated oxidative consumption rate also dominates over the production rate of lactate and alanine (*SI Appendix*, Table S1), indicating substantial oxidative metabolism.

### Labeling of Citrate with [2-^13^C]Pyruvate.

**Fig. 5.**
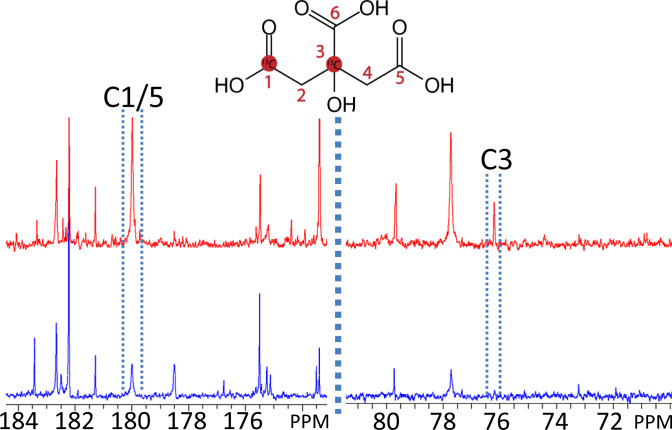
^13^C NMR spectra of growth medium of prostate cells after [2-^13^C]pyruvate supplementation. Spectra are shown of RPMI-1640 medium in which LNCaP cells (red) or VCaP cells (blue) have been grown for 48 h after supplementation of the medium with [2-^13^C]pyruvate. The positions of the C1 and C3 signals of citrate are indicated. Spectra are referenced and scaled to TSP at −2.0 ppm.

Pyruvate carbons can end up in citrate either through acetyl-CoA via PDHC (pyruvate dehydrogenase complex) or through oxaloacetate via PC, which provides an anaplerotic route to supply carbons to the Krebs cycle (Fig. 1 *A–E*). To estimate which fraction of pyruvate carbons enter the Krebs cycle via PC, we administered [2-^13^C]pyruvate instead of ^13^C-labeled glucose to both LNCaP and VCaP cells. This resulted in ^13^C label on the carbons C1/5 and C3 of citrate in the medium (Fig. 5).

When [2-^13^C]pyruvate is used to produce acetyl-CoA, the ^13^C label will end up at either C1 (first round Krebs cycle) or C5/6 (second round). However, if this ^13^C label enters the Krebs cycle via oxaloacetate (PC), it will end up at citrate C3 and C4 (first round in Krebs cycle) and half of this will end up in C3 and C4 (equally distributed, second round of the cycle). The carbons at C4 will go into C5 and C6 in the next round of the Krebs cycle and the carbons at C3 will be equally distributed over C3 and C4 again in the next round. C5 and C6 are lost as CO_2_. The amount of ^13^C label at position C1/5 was about 5.2 times higher than at position C3 for LNCaP and about 14 times higher for VCaP, which is an estimate of the fraction of pyruvate carbons flowing through acetyl-CoA relative to oxaloacetate.

In these experiments the fractional enrichment of lactate (measured from the ^13^C/^12^C ratio at the C2 position of lactate and alanine) in medium of LNCaP cells was estimated at 83%.

### Is Extracellular Citrate Labeled by Supplementation with [5-^13^C]Glutamine?

**Fig. 6.**
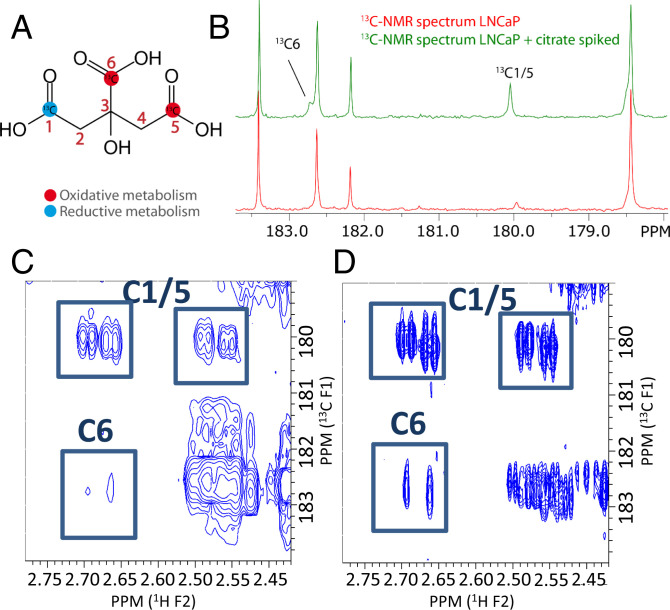
^13^C labeling of citrate after [5-^13^C]glutamine supplementation. (*A*) The ^13^C carbons of glutamine can label citrate at the C1 position (reductive metabolism) and at the C5 and C6 position (oxidative metabolism). (*B*) ^13^C NMR spectra of incubation medium of LNCaP cells (red) and of this medium spiked with citrate (green). The C1/5 and C6 resonances are indicated. (*C*) ^1^H-^13^C**-**HMBC heteronuclear correlation spectrum of the medium in *B*. Citrate resonances are indicated. The C6 resonances are resolved in the 2D spectrum from the overlapping resonance at 182.6 ppm. (*D*) ^1^H-^13^C**-**HMBC of incubation medium of VCaP cells supplemented with [5-^13^C]glutamine and incubated for 48 h. Citrate resonances are indicated. The very low level of citrate C6 signal confirms that most glutamine carbons ending up in citrate follow the reductive carboxylation pathway.

Finally, we tested whether glutamine could serve as carbon source for citrate secreted by LNCaP and VCaP. Glutamine carbons can enter the Krebs cycle via α-ketoglutarate produced from glutamate by GDH or by transamination and then follow an oxidative pathway via oxaloacetate or a reductive route via isocitrate and isocitrate dehydrogenase (IDH). The latter route may also be operational in the cytosol (Fig. 1*E*). Oxidative and reductive routes can both contribute ^13^C to secreted citrate when supplying the cells with [5-^13^C]glutamine, which will label citrate differently at the C1, C5, and C6 positions (Fig. 6*A*).

We observed that the ^13^C label of [5-^13^C]glutamine showed up in ^13^C-NMR spectra at carbon position C1/5 of citrate. Spiking one of the samples with the properly labeled citrate confirmed this assignment (Fig. 6*B*). Because the ^13^C carbon at position C6 overlaps with the resonance from pyroglutamate in 1D spectra we performed ^1^H-^13^C HMBC measurements to alleviate this overlap. As expected, the citrate C1/5 resonances were clearly visible, but also a C6 signal was observed (Fig. 6 *C* and *D*). These assignments were confirmed by spiking the samples with citrate (*SI Appendix*, Fig. S5).

The relative amount of label cannot be derived from the peak intensities in a ^1^H-^13^C HMBC spectrum directly, because of differences in J_CH_ coupling and relaxation. To determine the ratio of ^13^C label ending up in C1/5 and in C6, we recorded an additional ^1^H-^13^C HMBC spectrum on a sample of medium supplemented with unlabeled citrate. The known C1/5:C6 ratio (2:1) was compared to the found peak intensity C1/5:C6 ratio, giving a correction factor to derive the true C1/5:C6 citrate ratio in the [5-^13^C]glutamine labeling experiment. The presence of both ^13^C6 and ^13^C1/5 citrate after [5-^13^C]glutamine supplementation indicates that both reductive and oxidative pathways have been followed (*SI Appendix*, Table S3).

To estimate the contribution of C5 glutamine carbons to the C1,5,6 carbon pool in secreted citrate, the ^13^C-labeled citrate carbon concentration was determined by integrating the ^13^C1/5 resonance of citrate and using the ratio of reductive vs. oxidative metabolism, as calculated above, to estimate the total enriched citrate carbon integral (*SI Appendix*). Using the total amount of produced citrate, as determined by integrating the ^1^H resonances of citrate and normalizing this to the TSP resonance, the contribution of ^13^C from glutamine to citrate was estimated to be 26 to 29% (*SI Appendix*, Table S3). Since this labeling can be explained by exchange between the glutamate pool and the α-ketoglutarate, isocitrate, and citrate pool, this does not mean that glutamine carbons are an important source of carbon for citrate production, but rather that there are pathways available for this.

In the calculations above it was assumed that C5 and C6 in citrate are equally labeled. However, if labeled citrate is in fast exchange with isocitrate and α-ketoglutarate C6 labeling might be lost due to exchange with unlabeled carbon dioxide. The consequences of a reduced C6 labeling for these calculations as well as the possibility of citrate labeling via cytosolic reductive carboxylation are described in *SI Appendix*.

### Modeling Citrate Secretion and Relative Contribution of PC.

A unique property of prostate cells is their secretion of large amounts of citrate. Hence, it is of interest to explore if specific ^13^C-carbon labeling patterns of secreted citrate can be exploited to bear on metabolism inside prostate cells. For this purpose we developed a quantitative model to calculate measures of the divergence of citrate (and other molecules) from the Krebs cycle and the relative contributions of anaplerotic PC and pyruvate dehydrogenase complex. In this model the ^13^C labeling patterns of extracellular citrate after [1,6-^13^C_2_]glucose and [2-^13^C]pyruvate supplementation were used as simple input (*SI Appendix*).

The [1,6-^13^C]glucose added to the media is first converted to [3-^13^C]pyruvate during glycolysis before the label can enter the Krebs cycle via pyruvate dehydrogenase complex and acetyl-CoA and becomes incorporated in citrate at C2 (Fig. 1*B*). After completing one full Krebs cycle turn, this label ends up at the C2 or C3 position of oxaloacetate. If oxaloacetate then undergoes a condensation reaction with another ^13^C2-labeled acetyl-CoA, citrate is formed as 2,4-^13^C_2_ citrate or 2,3-^13^C_2_ citrate. The citrate resonances C2 and C4 show ^13^C–^13^C couplings with C3 and with each other. Resonances from differently ^13^C–^13^C-coupled C2 or C4 carbons cause the ^13^C signal of citrate centered at 46.6 ppm to be composed of different partly overlapping components (Fig. 4 and *SI Appendix*, Fig. S3). Next to label ending up in citrate via PDH, pyruvate label can also go into oxaloacetate directly via the PC reaction, this makes the label end up at citrate C3 and C4 (equally distributed due to fast exchange between the malate, fumarate and oxaloacetate pool). After one cycle, this label will be distributed over citrate C3, C4, C5, and C6. A fraction of citrate is secreted every Krebs cycle turn and depending on the number of cycles completed before secretion the ^13^C labeling pattern will be different. The sum of these contributions results in a specific ^13^C-labeling pattern of citrate in the medium. As this labeling pattern may be affected by other metabolites diverging from the Krebs cycle, we evaluate an apparent Krebs cycle secretion from the ^13^C distribution over the citrate carbons.

Because of the higher integrals of the C2+C4 and C3 signals of citrate, compared to those of C5 and C6, we propose a ratio *R*_1_ of these signals as a first measure of the apparent fraction of citrate molecules diverging from the Krebs cycle. We use this ratio as input for the model to calculate an apparent Krebs cycle secretion fraction *d* and relative contributions of the pyruvate dehydrogenase complex (PDC) and PC pathways (*SI Appendix*):

$$R_{1}=\frac{C_{2/4}-C_{3}}{C_{2/4}}.$$

Next to supplementation with 1,6-^13^C_2_–labeled glucose, which generates [3-^13^C]pyruvate, we applied [2-^13^C]pyruvate as an alternative substrate. The labels from [2-^13^C]pyruvate enter the Krebs cycle via acetyl-CoA and first end up in citrate C1 and after one cycle, in citrate C5 and C6. Carbon atoms going via PC to oxaloacetate will end up in citrate at C3 and C4 and after one cycle at C3, C4, C5, and C6 (Fig. 1 *B–E*). From these labeling patterns, we propose the ratio *R*_2_ to estimate the relative contribution of PC to the secreted citrate, since citrate C3 can only originate from ^13^C via PC (see Fig. 1 *B–E*):

$$R_{2}=\frac{C_{1/5}}{C_{3}}.$$

**Fig. 7.**
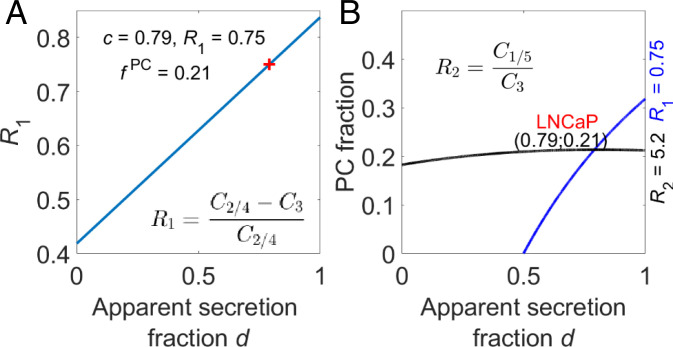
PC fraction vs. apparent secretion fraction *d* with experimental citrate carbon ratios R_1_ and R_2_. (*A*) *R*_1_ versus secretion fraction *d* after supplementation of growth media with [1,6-^13^C_2_]glucose. (*B*) Plot of PC fraction vs. secretion fraction *d* for experimentally found *R*_1_ = 0.75 and *R*_2_ = 5.2 after incubation of LNCaP in medium supplemented with [1,6-^13^C_2_]glucose or [2-^13^C]pyruvate for 48 h.

If the relative contribution of secreted citrate is large enough, *R*_1_ could be used as an index for m-aconitase inhibition and citrate secretion. If, for example, the fraction of secreted citrate dominates other cataplerotic pathways diverging carbons from the Krebs cycle the apparent Krebs cycle secretion fraction *d* approaches the real citrate secretion fraction *c* (*SI Appendix*). Then, most of it diverges at the first turn of the Krebs cycle and mainly C2 (PDH route) and C4 (PC route) citrates are present, making *R*_1_ ≈ 1 (Fig. 7*A*). If, on the other hand, carbon atoms of citrate are further processed in the Krebs cycle, about equal labeling of C2, C3 and C4 is expected and ratio *R*_1_ approaches 0.5. In this case the contribution of other cataplerotic pathways becomes more prominent (*c* < *d*). The offset at *d* = 0 in Fig. 7*A* is due to a contribution of the PC route.

The ratio *R*_1_ was found to be 0.74 and 0.76 for the two LNCaP samples, and the ratio *R*_2_ was estimated to be 5.2 ± 1.0 for LNCaP (*n* = 5) and 13.8 ± 1.4 (*n* = 2) for VCaP. Using both ratios as input for the quantitative metabolic model (*SI Appendix*), we calculated the apparent fraction of citrate to be secreted after every Krebs cycle turn to be 0.79 for LNCaP cells (Fig. 7*B*), which translates to an average number of 0.26 Krebs cycle turns before a label is secreted. Moreover, we calculated the fractional contribution of PC to the citrate production to be 0.21. In these calculations the specific ^13^C enrichment of pyruvate and acetyl-CoA was taken into account. Additional carbons leaving the Krebs cycle have no effect on the calculation of the fractional contribution of PC, though.

## Discussion

In this study we mapped metabolic pathways involved in the provision of carbons for citrate secreted by the human prostate cancer cell lines LNCaP and VCaP. For this purpose, we applied ^13^C-labeled substrates to track the incorporation of label in various carbon positions of citrate. The results of our experiments provide direct evidence that carbons of supplied glucose and glutamine are taken up in the carbon skeleton of citrate secreted by these cells. We identified glucose as the dominant carbon source for citrate synthesis and PC as the main anaplerotic route to compensate the Krebs cycle for the loss of carbons via secretion of citrate and other molecules. Further analysis of metabolic conversion rates indicates that glutamine carbons likely do not contribute to net synthesis of secreted citrate but can end up in this molecule by carbon exchange either oxidative via glutaminase or via reductive carboxylation. Labeled carbons of supplied aspartate or asparagine carbons do not end up in secreted citrate. Finally, a quantitative metabolic model was developed using the specific ^13^C labeling of secreted citrate as input to describe carbon flow from glucose and pyruvate to citrate.

Normal epithelial prostate cells have the unique capability to secrete high amounts of citrate. The current metabolic model to explain this property is that large quantities of Zn are taken up by these cells, which inhibits the mitochondrial enzyme m-aconitase (next to enzymes involved in respiration and terminal oxidation) and consequently leads to accumulation of citrate and to its secretion in prostatic lumen (18, 40). Because this inhibition quenches Krebs cycle activity, prostate epithelial cells have a relatively high glycolytic to oxidative phosphorylation (OXPHOS) balance (41). Upon transformation to malignancy, Zn uptake by these cells is down-regulated, which releases the inhibition of m-aconitase so that citrate is oxidized in the Krebs cycle at the cost of its secretion (9). We demonstrate that in LNCaP and VCaP cells, which are metastatic prostate cancer cells, the machinery to secrete citrate is still operational. This is likely due to their relatively differentiated nature: Both are androgen-responsive and produce PSA (29, 42). Less-differentiated cells like PC3 and DU145 do not produce citrate for secretion (27). However, citrate production by LNCaP is 10 times higher than by VCaP, which could be related to the metastatic origin of VCaP (bone) as compared to that of LNCaP (lymph node), suggesting that VCaP is less differentiated (29, 42).

As the zinc transporter hZIP1 is still present in LNCaP cells (5, 43, 44) we tested the effect of adding Zn^2+^ to the incubation medium. Citrate production was either decreased (LNCaP) or unaffected (VCaP). This may be because the cells already contained Zn coming from the fetal bovine serum they were originally grown in (5, 45) and any additional Zn^2+^ does not further enhance citrate production. Zinc can be toxic to these cells (40, 41), but at a concentration of 50 µM we observed no cell death, as reported previously (46). Still, zinc can have multiple inhibitory effects on malignant cells, like LNCaP, such as on mitochondrial and glycolytic enzyme activity (47–49). Indeed, the lower levels of secreted citrate, lactate, and alanine and increased alanine/lactate indicate altered metabolic activity in LNCaP cells after Zn addition, possibly associated with Zn storage (50).

Alternatively, despite the presence of hZIP1, zinc uptake and inhibition of oxidative processes may not be very effective in LNCaP cells, which is in line with the relatively high estimated oxidative rate we observed for these cells. The proposed metabolic progression of malignancy in prostate epithelial cells first involves an up-regulation of OXPHOS with respect to glycolysis, followed by a switch to a Warburg profile in more aggressive cancer cells (40, 41, 51). LNCaP cells still might be in a more oxidative phase, in agreement with a study indicating that parental LNCaP cells have oxidative properties (52).

As citrate in normal epithelial cells diverges from the Krebs cycle, anaplerotic carbon provision of this cycle is needed. Studies of rat prostate epithelial cells indicated that aspartate supplementation is required for citrate production and thus that conversion of aspartate into oxaloacetate may function as an anaplerotic route (20). This view was supported by the finding that the aspartate–glutamate transporter EAAC1 is widely present in human prostate epithelial cells (23). As this also included LNCaP cells, we expected that addition of ^13^C-labeled aspartate to the incubation medium of LNCaP and VCaP cells would result in ^13^C labeling of citrate (21, 22). However, no detectable ^13^C resonance for citrate was observed in spectra of the incubation media, despite the presence of clear citrate peaks in ^1^H NMR spectra of the same samples. We also did not observe ^13^C aspartate resonances in extracts of LNCaP and VCaP, which indicates that their uptake of aspartate is low or absent despite the presence of transporters for aspartate (23). Similar negative results were obtained when replacing aspartate by asparagine. Interestingly, aspartate addition to the medium of LNCaP androgen-ablated cells was not able to rescue the effect of mitochondrial pyruvate carrier inhibition, while glutamine and glutamate were able to do this (53). Apparently, aspartate or asparagine is not an absolute requirement for Krebs cycle production of citrate by human prostate cancer cells, but we cannot exclude that they have a role in citrate production by normal prostate epithelial cells.

To further investigate which compounds can supply carbon atoms for secreted citrate in our human cell lines, we incubated them with either ^13^C-labeled glucose, pyruvate, or glutamine. As ^13^C-enriched citrate was observed in the medium in all cases, this identifies these compounds as potential carbon sources for secreted citrate. The labeling patterns observed in these experiments can only occur when the carbon atoms participate in multiple turns of the Krebs cycle, which is further direct evidence that m-aconitase, the enzyme converting citrate into isocitrate, is active. In our study, LNCaP grew much faster than VCaP, in agreement with its four-times-higher glucose consumption rate.

The significant generation of ^13^C-labeled citrate after supplementation of LNCaP and VCaP cells with [^13^C]glucose or [^13^C]pyruvate identifies glycolysis as a main route to supply carbons for citrate secreted by these cells. Interestingly, glucose also stimulates Zn release by normal human prostate epithelial cells (54). The higher ^13^C labeling at citrate C1 vs. C3 after incubation with [2-^13^C]pyruvate indicates that the majority of these carbon atoms enter the Krebs cycle via the pyruvate dehydrogenase (PDC) reaction and acetyl-CoA. From the ratio $R_{2}=C_{1/5}/C_{3}$ and the carbon distribution model, we estimated that the fraction of pyruvate carbons flowing to the Krebs cycle through the PC reaction is about 0.21 for LNCaP and 0.12 for VCaP. In this context it is striking that VCaP produces less citrate than LNCaP. PC activity varies greatly in tumors, which may represent adaptations to the specific needs for anaplerosis in each tumor type (55–57).

Supplementation with [5-^13^C]glutamine shows that carbons for citrate secreted by LNCaP and VCaP also originate from glutamine. In proliferating cancer cells, glutamine is known to be a major anaplerotic precursor, and increased glutaminolysis is recognized as a key feature of metabolism in these cells (58–62). After  conversion of glutamine into α-ketoglutarate its carbons can end up in citrate in mitochondria via oxidative metabolism or via a reversed Krebs cycle by reductive carboxylation through IDH and aconitase (63–65). Reductive carboxylation can also proceed in the cytosol via IDH1 and aconitase-1 reactions, as was shown to be operational in several cell lines (63, 65). According to our HMBC experiments about 60% of ^13^C observed in citrate after glutamine supplementation followed a reductive carboxylation pathway in LNCaP and VCaP and the other part proceeded via an oxidative Krebs cycle route. However, as reductive carboxylation involves exchange reactions the ^13^C observed via this route may come from isotope exchange and not net synthesis of citrate (66). In the presence of glucose, glutamine accounts for 20 to 25% of the carbons present in extracellular citrate in LNCaP and VCaP. Despite this labeling our comparison of PC and citrate secretion rates indicate that glutamine carbons are not an absolute requirement for anaplerosis to compensate for citrate secretion under the current circumstances.

Interestingly, reductive carboxylation seems particularly important in cancer and other cells with an impaired Krebs cycle (64, 67, 68). As this cycle in normal prostate epithelial cells is impaired, it is plausible that citrate synthesis in these cells not only occurs in mitochondria in the first step of the Krebs cycle but also is supported by carbon flow via reductive carboxylation of glutamine mediated by IDH and aconitase (Fig. 1*A*).

Although oxidative phosphorylation appears to be restricted in epithelial prostate cells the extent to which Krebs cycle activity is reduced is not known. The quantitative model described in *SI Appendix* provides an estimate of the apparent Krebs cycle secretion fraction per average number of full Krebs cycle turns. In this estimation we used two simple ratios of ^13^C signal integrals of NMR spectra obtained after supplementation with ^13^C-labeled glucose or pyruvate. This measure is independent of ^13^C enrichment of the administered supplements of which carbons enter the Krebs cycle, independent of ^13^C J-coupling patterns and can be easily adjusted for use with other ^13^C-labeled supplements. Along with this Krebs cycle extraction number, we present an estimation of the fractional contribution of PC and PDC to carbons entering the Krebs cycle. The apparent secretion fraction *d* calculated from the model may not only represent secretion of citrate but also the efflux of other metabolites during each cycle turn, which makes the secretion fraction *d* an upper limit for the true citrate secretion fraction *c*. Other secretion pathways like GDH, aspartate transaminase, PEPCK, and malic enzymes can catalyze the efflux of carbons from the Krebs cycle. The latter two enzyme reactions can contribute to pyruvate production and thus may be responsible for the observed dilution of ^13^C in pyruvate to 80% enrichment after supplementation with 99% [1,6 ^13^C]glucose and [2-^13^C]pyruvate. The excess anaplerotic provision of PC, which we estimated in this study, may compensate for the loss of Krebs cycle carbons by these effluxes, such as carbons of glutamate.

Multiple papers on metabolic flux models report high exchange fluxes associated with α-ketoglutarate–glutamate conversion, comparable to total Krebs cycle fluxes, from in vivo studies in tissues like the heart (69, 70) and brain (71–74) and from in vitro studies in melanoma (75), hepatocyte (76), and glioma (58) cells. Depending on the size of the unlabeled glutamate pool this can influence the loss of ^13^C labels to other metabolic networks and partially account for the efflux of labeled carbons from the Krebs cycle. Until now no literature values for such exchange fluxes are available for prostate. Future efforts can be made to extend the model with an estimation of intracellular glutamate or other abundant metabolites facilitating Krebs cycle outflow to better estimate the real citrate secretion fraction per number of cycles.

Next to citrate, other labeled metabolites were identified in the medium by 2D ^1^H-^13^C correlation spectroscopy. These included [3-^13^C]lactate and [3-^13^C]alanine that are produced in the cytoplasm by glycolysis from [1,6-^13^C_2_]glucose via [3-^13^C]pyruvate. Halfway through glycolysis, ^13^C-labeled dihydroxyacetone can be converted into [1/3-^13^C]glycerol via *sn*-glycerol-3-phosphate and ^13^C-labeled glycerate-3-phopshate can be converted via serine into [2-^13^C]glycine. High rates of synthesis and consumption of glycine have been found to be correlated with rates of tumor cell proliferation in 60 tumor cell lines, among which PC3, and uptake and secretion rates were determined showing different rates of secretion of these metabolites (77). If ^13^C label enters the Krebs cycle via acetyl-CoA, it can be incorporated in [4-^13^C]glutamate via α-ketoglutarate. Glutamate can subsequently be converted into [4-^13^C]proline via Δ^1^-pyrroline-5-carboxylate, a process that has been shown to be up-regulated by oncogenic transcription factor c-MYC in prostate cancer cell line PC3 (78). These metabolites are also secreted by other cells, such as nonsmall-cell lung cancer cell lines H1299 and A549 (79).

In conclusion, we identified a number of carbon sources and metabolic pathways contributing to carbons in secreted citrate in human prostate cancer cells LNCaP and VCaP. Although metabolism of healthy epithelial cells is expected to be different, essentially these sources and pathways are also available in these cells and thus are potential candidates to maintain the citrate pool in the lumen of the normal prostate. After application of ^13^C substrates the ratios of ^13^C carbons in extracellular citrate may serve as biomarkers to report on (malignant) alterations in epithelial cell metabolism.

## Materials and Methods

### Cell Lines.

The cell lines LNCaP (ATCC CRL-1740) and VCaP (ATCC CRL-2876), both derived from human metastases of prostate adenocarcinoma, were a gift from Gary J. Miller, University of Colorado Health Sciences Center, Denver, CO. The cells were grown in full RPMI-1640 medium supplemented with glutamine (2 mM), fetal calf serum (10% vol/vol), 100 U/mL penicillin, and 100 µg streptomycin (LNCaP passage number was ∼48). Approximately 5 × 10^6^ to 15 × 10^6^ LNCaP cells or 15 × 10^6^ to 20 × 10^6^ VCaP cells were used per flask in the ^13^C-labeling experiments. For the citrate production rate experiments, ∼30 × 10^6^ LNCaP cells or 40 to 50 × 10^6^ VCaP cells were used.

### ^13^C Labeling.

When the number of cells was large enough (i.e., when full confluency was achieved), the growth medium was replaced by fresh RPMI-1640 medium supplemented with glutamine and different ^13^C-labeled substrates. No serum was added during this incubation to avoid broad baselines in NMR spectra due to the high protein content of serum, unless indicated otherwise. The cells were incubated for 48 h at 37 °C, 5% CO_2_. In all experiments, a low concentration of aspartate (150 µM) was present in the standard formulation of RPMI-1640 (80).

Both LNCaP and VCaP were grown in medium in which glucose (11 mM) was replaced with 99% enriched [1,6-^13^C_2_]glucose (11 mM). In different experiments, [U-^13^C_4_]aspartate (2.0 mM) or [U-^13^C_4_]asparagine (2.0 mM) were added to test both aspartate and asparagine as carbon sources, in which the standard unlabeled glucose was present, or it was replaced by pyruvate (6 mM). [5-^13^C]glutamine (2mM) and [2-^13^C]pyruvate (7 mM) were also tested as carbon sources for secreted citrate. In the experiments with ^13^C-labeled glutamine, it replaced unlabeled glutamine. In the experiment with ^13^C-labeled pyruvate, medium without glucose was used. In all experiments, glutamine and glucose concentrations were the same, unless glucose was replaced by pyruvate. All ^13^C-labeling experiments were done in duplicate and one additional flask per experiment was supplemented with the unlabeled equivalent of the corresponding ^13^C substrates. Supplementations with these substrates were used to analyze ^13^C-labeling patterns in secreted citrate (Fig. 1 *B–E*).

### Sample Preparation.

After 48 h the media were collected and immediately placed on ice. Detached cells possibly present in the media were removed by centrifugation (3,750 rpm, 2 °C, 10 min). Next, 4-mL aliquots of the media were taken and TSP was added as NMR reference with a final concentration of 0.2 mM. Subsequently, the samples were frozen before lyophilization overnight.

For every experiment, one of two flasks containing ^13^C-labeled substrates was used to estimate the number of cells. For this purpose, after collecting the medium the cells were washed with phosphate-buffered saline (PBS) buffer and isolated by adding a trypsin solution. After a short incubation period (37 °C, 5 min), the cells were dissolved in fresh complete RPMI-1640 buffer and counted using a Neubauer counting chamber. The cells in the remaining two flasks (one ^13^C-labeled, one unlabeled) were extracted to check for the presence of ^13^C-labeled substrates and other metabolites. Cells were washed with PBS buffer twice before adding a methanol–water mixture (1:1). The cells were detached using a scraper and the cell extract was collected. The cell extracts were shaken vigorously before they were placed on ice. Subsequently, the cell extracts were centrifuged (3,750 rpm, 2 °C, 10 min) to remove the water-insoluble parts. The supernatant was collected and the methanol was removed using a Savant SpeedVac concentrator. The samples were then frozen before lyophilization overnight.

### NMR Spectroscopy.

Lyophilized samples were dissolved in 500 µL D_2_O, the pH adjusted to 7.4, and analyzed on Bruker Avance and Avance III 500 MHz spectrometers. Water-suppressed ^1^H NMR spectra were recorded with 256 to 320 scans for medium samples and 640 scans for cell extract samples. ^13^C NMR spectra were recorded with 5,000 to 21,000 scans using WALTZ64 ^1^H-decoupling. Assignment of the citrate resonances was confirmed by spiking samples after initial measurements. In addition, 2D ^1^H-^13^C HSQC and HMBC correlation experiments were performed for all labeling experiments to confirm resonance assignments and to check for the presence of citrate and other ^13^C-labeled metabolites. ^1^H-^13^C HMBC experiments were performed on the [5-^13^C]glutamine–supplemented samples to separate overlapping resonances and additionally on one fresh medium sample spiked with citrate as a reference for the glutamine experiments, to compare peak intensities to those in labeled experiments. Further, ^1^H-^13^C correlations were measured in the LNCaP media supplemented with [1,6-^13^C_2_]glucose to determine the presence of other ^13^C-labeled metabolites.

### Estimation of Citrate Production and Zinc Supplementation.

Six flasks of both LNCaP and VCaP cells were incubated in RPMI-1640 + 2 mM Gln + S/P without serum (LNCaP) or with 2% carbon-stripped serum (VCaP; these cells detach from the flask without serum) and grown to full confluency. As described above the medium was replaced by fresh medium; for three flasks per cell line this contained 50 µM Zn^2+^, added from a ZnCl_2_ solution, and three flasks received medium without added Zn(II). After 48 h the medium was collected and the cells were harvested and counted. Aliquots of 4 mL medium were lyophilized after addition of TSP and dissolved in D_2_O for ^1^H NMR. The ^1^H peak integrals of citrate, lactate, and alanine and TSP as reference were used to estimate their average production over 48 h. Independent two-sample *t* tests were performed to determine if Zn^2+^ addition to the medium significantly changed the production of citrate, lactate, or alanine.

### ^13^C Enrichment of the Glucose Pool after Supplementation with [1,6-^13^C_2_]Glucose.

Since intracellular glucose is still present when the unlabeled medium is replaced by medium supplemented with [1,6-^13^C_2_]glucose, we estimated the ^13^C enrichment of the glucose pool after supplementation by calculating the integral ratio of ^12^C-lactate and ^13^C-lactate in the ^1^H NMR spectra of the growth media after 48 h, assuming the rate of lactate production is the same for ^13^C-labeled glucose and unlabeled glucose. To estimate the relative production of lactate and citrate over 48 h originating from ^13^C-labeled glucose, the ^13^C-lactate and ^13^C-citrate signals in ^13^C NMR spectra of the incubation media were integrated to calculate their ratio.

### Fitting of ^13^C-Labeled Citrate Signals after [1,6-^13^C_2_]Glucose Supplementation.

The signals of ^13^C-labeled citrate were fitted using AMARES in software program *j*MRUI (81–83), with a singlet for C2 and C4 at 46.5 ppm and a doublet for C2–C3. We assumed Lorentzian lineshapes and linewidths were constrained between 4 and 20 Hz. We assumed the same phase for all peaks and fitted the two smaller J-coupled peaks in the C2 region at δ(^13^C2)+19 Hz and δ(^13^C2)−19 Hz (assuming ^1^J^C2C3^ = 38 Hz). The J-coupling between C2 and C4 is smaller than the ^13^C linewidth and was therefore neglected in the fit. In the C3 region at 76.2 ppm, the chemical shift of the four outer peaks were set to δ(^13^C3)−38 Hz, δ(^13^C3)−19Hz, δ(^13^C3)+19 Hz, and δ(^13^C3)+38 Hz with respect to the central C3 peak.

## Supplementary Material

Supplementary File

## Data Availability

All data used for this study are deposited in the publicly accessible Zenodo repository at https://doi.org/10.5281/zenodo.5752953.
