# Supplementary material for: Carbon sources and pathways for citrate secreted by human prostate cancer cells determined by NMR tracing and metabolic modeling
Source: Proc Natl Acad Sci U S A. 2022 Mar 30;119(14):e2024357119. doi: 10.1073/pnas.2024357119 (PMC9168453; doi:10.1073/pnas.2024357119)
Supplement: Supplementary File [file pnas.2024357119.sapp.pdf]

PNAS
www.pnas.org

Supplementary Information for

Carbon sources and pathways for citrate secreted by human prostate cancer cells determined by NMR tracing and metabolic modeling

Frits H.A. van Heijster, Vincent Breukels, Kees (C).F.J. Jansen, Jack A. Schalken, Arend Heerschap

Arend Heerschap
Email: Arend.Heerschap@radboudumc.nl

This PDF file includes:

Supplementary text

- Mass balance evaluation to estimate pyruvate carboxylase flux
- Calculation of the ratio of net ^{13}C-citrate produced via reductive exchange vs. oxidative metabolism after [5-^{13}C]glutamine supplementation.
- Calculation of the fraction of ^{13}C in extracellular citrate arising from [5-^{13}C]glutamine
- Calculation of the dilution of the ^{13}C-acetyl-CoA pool
- Quantitative model describing production of extracellular citrate from glucose and pyruvate

Figs. S1 to S6

Tables S1 to S5

Supplementary Information Text

Mass balance evaluation to estimate pyruvate carboxylase flux

For the estimation of the pyruvate carboxylase flux we consider the following balance in flux rates in the LNCaP cells:

$v_{GU} = v_{Sec,\,NMR} + v_{Sec,\,other} + v_{BM} + v_{OX,,}$ in which

v_{GU} is the glucose uptake rate (Table S2),

$v_{Sec,\,NMR}$ is the metabolite secretion rate of alanine, lactate and citrate (Table S1),

$v_{Sec,\,other}$ is the metabolite secretion rate of glycerol, acetate, and glycine,

v_{BM} is the biomass generation rate of pathways prior to the pyruvate kinase reaction (e.g. ribose, serine, glycerol-3-phosphate),

v_{OX} is the oxidative flux rate including all reactions that follow the PDH reaction (except citrate secretion), also including glutamate and proline secretion.

If we express the rates in terms of triose units (i.e. glucose and citrate are 2 trioses, while the other compounds are 1 triose) per nmol/h/10^6 cells we arrive at the following expression '

$2 \times 254 = (147 + 21 + 2 \times 5.6) \; + v_{Sec,\,other} + v_{BM} + v_{OX}$

Or $329 = + v_{Sec,\,other} + v_{BM} + v_{OX}$

If we assume that ~ 20% of trioses from glucose goes to compounds for $v_{Sec,\,other}$ *and* v_{BM}

then $v_{OX} \approx 280$ nmol/h/10^6 cells

and given that $v_{PC} / v_{PDH} = 0.21$ this results in an estimated $v_{PC} \approx 58$ nmol/h/10^6 cells.

In triose units this is about 5 times larger than the production rate of secreted citrate (~11 nmol/h/10^6 cells).

Calculation of the ratio of net ^{13}C-citrate produced via reductive exchange vs. oxidative metabolism after [5-^{13}C]glutamine supplementation.

^{13}C-carbons from [5-^{13}C]glutamine supplied to the tumor cell lines can end up in citrate in the mitochondria, after conversion via glutamate to α-ketoglutarate, via reductive carboxylation and/or via forward (oxidative) Krebs cycle metabolism [1]. However, as the reductive carboxylation pathway concerns exchange between α-ketoglutarate, isocitrate and citrate [2] we have labeled this pathway as reductive exchange. To calculate the fraction of carbons using either of these two routes of citrate labeling in the [5-^{13}C]glutamine experiments we start by defining f_{Krebs} and f_{exch} as the fractions of net produced labeled citrate via these routes (the reductive carboxylation / exchange pathway can also occur in the cytosol, this is discussed below). Note that these fractions do not correspond to absolute fluxes through oxidative or reductive pathways, since exchange between alpha-ketoglutarate and citrate on one hand and possible dilution of labeled molecules during later steps in the oxidative pathway underestimate both fluxes to a different extent. Net produced citrate via oxidative or reductive metabolism can however be distinguished. When carbon-13 coming from glutamine follows the oxidative pathway towards citrate, part of the molecules, f_{dil}, might be lost due to dilution by unlabeled molecules

flowing into the Krebs cycle after the irreversible conversion of alpha ketoglutarate into succinyl-CoA (*e.g.* unlabeled succinate, malate, fumarate). The sum of these fractions has to be equal to 1.

$$f_{Krebs} + f_{exch} + f_{dil} = 1 \quad \textit{[Equation S1]}$$

$C_{n.a.} = [Unlabeled\ citrate]$, concentration of unlabeled citrate. *[Equation S2]*

$C_{exp} = [Labeled\ citrate]$, concentration of labeled citrate. *[Equation S3]*

The NMR signal I_{C_i} recorded for carbon C_i (all carbons mentioned here are ^{13}C) will be proportional to the concentration $[C_i]$ and pulse sequence-dependent variables combined in bulk variable ε, which can be different for different carbons in more complicated pulse sequences (e.g. ^1H-^{13}C HMBC, the individual NMR signals scale with the size of the J-coupling), but assumed here to be constant for different samples for which only concentration $[C_i]$ varies over the different experiments but for which the *same pulse sequence* was used:

$$I_{C_i} = \varepsilon_{NMR}[C_i] \quad \textit{[Equation S4]}$$

[5-^{13}C]glutamine carbons converted into α-ketoglutarate end up in citrate at C1 via exchange, while carbons following the oxidative pathway will end up in citrate divided over C5 and C6. Part of the C6 label may be lost as carbon dioxide because of the exchange with isocitrate and α-ketoglutarate, so we introduced a loss fraction ϵ_c. The dilution of the Krebs cycle metabolites by inflow of unlabeled metabolites (f_{dil}) is assumed to be 0 for the calculation and is discussed below.

$$\begin{cases} [C_1]_{exp} = Cf_{exch} \\ [C_5]_{exp} = \frac{C}{2}f_{Krebs} \\ [C_6]_{exp} = \epsilon_c\frac{C}{2}f_{Krebs} \end{cases} \quad \textit{[Equation S5]}$$

With $[C_1]_{exp}$ the net produced citrate via the reductive exchange pathway and $[C_5]_{exp}$ and $[C_6]_{exp}$ the net produced citrate via Krebs cycle metabolism. Noteworthy here is that since citrate produced via reductive metabolism can also be converted back into alpha-ketoglutarate by exchange, only net production is seen here. No information about fluxes via reductive or oxidative metabolism can be extracted. If we evaluate the ratio between the experimental C1+C5 concentrations (their signals have equal chemical shift) and the C6 concentration we arrive at the following equation:

$$\frac{[C_1]_{exp}+[C_5]_{exp}}{[C_6]_{exp}} = \frac{f_{exch}+\frac{f_{Krebs}}{2}}{\epsilon_c\frac{f_{Krebs}}{2}} = \frac{1}{\epsilon_c}\left(1 + 2\frac{f_{exch}}{f_{Krebs}}\right) \quad \textit{[Equation S6]}$$

Now we can derive an expression for the ratio *E* of NMR signal integrals of C_1+C_5 and C_6 using Equation S4. Since C1 and C5 are equivalent in the HMBC experiment (J-couplings are also the same) we can use the same ε_1 for both carbons, but need to use a different ε_2 for C_6 (different J-couplings).

$$E = \left(\frac{I_{C_1}+I_{C_5}}{I_{C_6}}\right)^{HMBC}_{lab.\ citr.} = \frac{\varepsilon_1}{\varepsilon_2}\frac{[C_1]_{exp}+[C_5]_{exp}}{[C_6]_{exp}} \quad \textit{[Equation S7]}$$

This ratio $\varepsilon_1/\varepsilon_2$ will be equal in an experiment in which all carbons are equally enriched, so we also measured a medium sample with natural abundance citrate added. Of all carbons in unlabeled citrate 1.1% are ^{13}C.

$$[C_1]_{n.a.} = [C_5]_{n.a.} = [C_6]_{n.a.} = 0.011C_{n.a.} \quad \textit{[Equation S8]}$$

Let's introduce a ratio similar to *E* for natural abundance citrate:

$$U = \left(\frac{I_{C_1}+I_{C_5}}{I_{C_6}}\right)^{HMBC}_{n.a.\ citrate} = \frac{\varepsilon_1[C_1]_{n.a.}+\varepsilon_1[C_5]_{n.a.}}{\varepsilon_2[C_6]_{n.a.}} = \frac{2\varepsilon_1}{\varepsilon_2} \quad \textit{[Equation S9]}$$

With U we have the ratio of signal integrals for $(C_1+C_5)/C_6$ for an unlabeled citrate sample. Now we can substitute $\frac{\varepsilon_1}{\varepsilon_2} = \frac{U}{2}$ in Equation S7 and combine with Equation S6 to get:

$$E = \frac{U}{2}\frac{[C_1]_{exp}+[C_5]_{exp}}{[C_6]_{exp}} = \frac{U}{2\epsilon_c} + U\frac{f_{exch}}{f_{Krebs}\epsilon_c} \qquad \textit{[Equation S10]}$$

Now we can calculate the fraction of labeled citrate produced from glutamine via exchange vs. citrate produced via oxidative Krebs cycle metabolism from the two integrals defined above.

$$\frac{f_{exch}}{f_{Krebs}} = \frac{E}{U}\epsilon_c - \frac{1}{2} \qquad \textit{[Equation S11]}$$

The experimental ratios E and U were determined for LNCaP and VCaP tumor cells (Table S3). If the dilution fraction is $\epsilon_c=1$ (fast secretion, no dilution in the isocitrate and α-ketoglutarate pool), we can calculate an f_{exch}/f_{Krebs} of about 1.4 for LNCaP and 2.0 for VCaP. If on the other hand dilution of the C6 label was fast ($\epsilon_c \approx 0$), no citrate C6 signal would have been visible in the spectra. In the cases in between, the calculated values are an overestimation of the real reductive exchange vs. Krebs cycle metabolism. Another consideration is the dilution of oxidatively produced citrate by inflow of unlabeled metabolites like succinate or malate. We assume this to be small compared to the large dilution of the label in the glutamate pool. The latter has the same effect on both reductive and oxidative metabolism and does not change the outcome of the calculations.

The citrate labeling patterns found in the medium are a read-out of the labeling pattern in the cytosol of the cells, just before secretion. In the calculation above we did not consider yet the possible difference in production of labeled citrate via reductive carboxylation in the cytosol and in the mitochondria. Citrate produced in the cytosol (only by the reductive exchange route) directly ends up in the cytosol citrate pool. In principle citrate carbons can be shuttled between cytosol and mitochondria, but this would occur via multi-reaction pathways of which the relevance in prostate epithelial cells has not been demonstrated yet and assumed to be negligible and not considered here. Citrate in the mitochondria produced via either reductive exchange or oxidative Krebs cycle metabolism, only ends up partially in the cytosolic citrate pool because part of this citrate stays in the Krebs cycle. If we follow the calculation that about 80% of citrate is diverged from the Krebs cycle (see section on the quantitative model below), this means that 20% of labeled citrate via these pathways is not ending up in the citrate pool in the cytosol. This has an effect on the fraction of labeled citrate produced in the different pathways. If all citrate is produced in the mitochondria, the ratio of citrate produced via oxidative Krebs cycle metabolism vs. reductive exchange is simply the fraction we calculated above (for LNCaP $f_{exch}/f_{Krebs} = 1.41$ and for VCaP $f_{exch}/f_{Krebs} = 2.03$):

$$\left(\frac{f_{exch}}{f_{Krebs}}\right)_{real} = \left(\frac{0.8 \cdot f_{exch}}{0.8 \cdot f_{Krebs}}\right)_{exp} \qquad \textit{[Equation S12]}$$

If (in the other limit) all labeled citrate produced in the mitochondria goes via oxidative Krebs cycle metabolism, and all labeled citrate produced via reductive carboxylation is produced in the cytosol, only the fraction proceeding via oxidative metabolism has to be multiplied by 0.8.

$$\left(\frac{f_{exch}}{f_{Krebs}}\right)_{real} = \left(\frac{0.8 \cdot f_{exch}}{f_{Krebs}}\right)_{exp} \qquad \textit{[Equation S13]}$$

This changes the ratio for LNCaP to f_{exch}/f_{Krebs} = 1.13 and for VCaP to f_{exch}/f_{Krebs} = 1.62. The real ratio between citrate produced from glutamine via net oxidative and citrate produced via net reductive metabolism will be in between, so for LNCaP f_{exch}/f_{Krebs} = 1.13-1.41 and for VCaP f_{exch}/f_{Krebs} = 1.62-2.03.

Calculation of the fraction of ^{13}C in extracellular citrate arising from [5-^{13}C]glutamine.

To calculate the fraction of citrate labeled by [5-^{13}C]glutamine (i.e. $^{13}C/(^{13}C+^{12}C)$) from 1D ^{13}C NMR we first have to integrate all its carbon resonances. If the signal-to-noise ratio of the ^{13}C NMR spectrum is not sufficient to integrate all peaks or if there is too much overlap with other resonances, we can use the estimate of the ratio of reductive exchange and oxidative metabolism as calculated above to estimate the summed integral of all carbons if we have at least one of the individual integrals. For example if we know the integral of the C_1+C_5 citrate signals from a 1D ^{13}C NMR experiment, but we don't know the integral of C_6, we can use the Equations S4-S6 and the fact that we can get the same factor ε_3 for all carbons in a simple 1D ^{13}C experiment and using a reference with known concentration we can calculate the ^{13}C-labeled citrate concentration.

$$I_{ref} = \varepsilon_3[C_{ref}] \Rightarrow \varepsilon_3 = \frac{I_{ref}}{[C_{ref}]} \quad \textit{[Equation S14]}$$

$$Q = \left(\frac{I_{C_1}+I_{C_5}}{I_{C_6}}\right)^{1D}_{lab.\ citr.} = \frac{\varepsilon_3([C_1]_{exp}+[C_5]_{exp})}{\varepsilon_3[C_6]_{exp}} \quad \textit{[Equation S15]}$$

$$\left(\frac{I_{C_1}+I_{C_5}}{I_{C_6}}\right)^{1D}_{lab.\ citr.} = 1+2\frac{f_{exch}}{f_{Krebs}} \Rightarrow \left(I_{C_6}\right)^{1D}_{lab.\ citr.} = \frac{\left(I_{C_1}+I_{C_5}\right)^{1D}_{lab.\ citr.}}{1+2\frac{f_{exch}}{f_{Krebs}}} \quad \textit{[Equation S16]}$$

$$[C_1]_{exp}+[C_5]_{exp}+[C_6]_{exp} = \frac{\left(I_{C_1}+I_{C_5}\right)_{lab.\ citr.}}{\varepsilon_3}\left(1+\frac{1}{1+2\frac{f_{exch}}{f_{Krebs}}}\right) \quad \textit{[Equation S17]}$$

$$[C_1]_{exp}+[C_5]_{exp}+[C_6]_{exp} = [C_{ref}]\frac{\left(I_{C_1}+I_{C_5}\right)_{lab.\ citr.}}{I_{ref}}\left(1+\frac{1}{1+2\frac{f_{exch}}{f_{Krebs}}}\right) \quad \textit{[Equation S18]}$$

So, if we calculate the concentration of $^{13}C1/5$ from an NMR experiment using a known reference and subsequently multiply with the factor $\left(1+\frac{1}{1+2\frac{f_{exch}}{f_{Krebs}}}\right)$ we have an estimate of the real concentration of labeled citrate coming from [5-^{13}C]glutamine. Since the estimate of ^{13}C ending up in citrate via net reductive metabolism vs. net Krebs cycle metabolism is an overestimation, the ^{13}C-labeled citrate produced from ^{13}C-labeled glutamine found in this manuscript most likely is an underestimation. Note here that a large contribution of ^{13}C-carbons from glutamine to citrate can be explained by fast exchange of glutamate carbons into the Krebs cycle pool, and do not necessarily imply a large contribution of glutamine to citrate production.

Calculation of the dilution of the ^{13}C-acetyl-CoA pool

To calculate the ^{13}C enrichment of the acetyl-CoA pool the glutamate C3 singlet peak in the medium is compared to the doublet peak of C3 spins that are coupled to the C4 spins. Glutamate will be labeled at the C4 position during the first Krebs cycle turn when the cells are supplemented with [1,6-$^{13}C_2$]glucose and half of this will end up at C3 during the

next round. Depending on the enrichment of the acetyl-CoA pool this will result in a doublet (double labeling at C3 and C4) or in a single label (singlet at C3). This means the ^{13}C-enrichment of the acetyl-CoA pool can be determined by calculating [d(C3C4]/[s(C3)+d(C3C4)]. In these calculations, the natural abundance of ^{13}C in C3 and C4 should be considered. Since no carbon-13 ends up at position C5, the intensity of this peak can be used to compensate for natural abundance carbon-13. In absence of a significant $^{13}C5$ peak, this was omitted in our calculations.

References

1. Altman BJ, Stine ZE, and Dang CV. From Krebs to Clinic: Glutamine Metabolism to Cancer Therapy. Nat Rev Cancer. 2016 October ; 16(10): 619–634.
2. Fan J, Kamphorst JJ, Rabinowitz JD, Shlomi T. Fatty acid labeling from glutamine in hypoxia can be explained by isotope exchange without net reductive isocitrate dehydrogenase (IDH) flux. J Biol Chem. 2013 Oct 25;288(43):31363-9.

Quantitative model describing the production of extracellular citrate from glucose and pyruvate

This section describes the quantitative model that we developed for the calculation of two metabolic parameters reported in the main text. Firstly, this model has as output a measure of cellular secretion of citrate or other molecules (citrate equivalents), in the form of an apparent Krebs cycle secretion fraction d of molecules that leave the Krebs cycle every cycle turn. Secondly, it provides the relative contribution of pyruvate carboxylation f^{PC} and of pyruvate dehydrogenase complex f^{PDC} routes to citrate production. Prostate epithelial cells have the unique capability to secrete large amounts of citrate. Our model uses as input the specific ^{13}C labeling pattern of this citrate, secreted by LNCaP cells in the medium after administration of ^{13}C labeled substrates, specifically [1,6-$^{13}C_2$]glucose and [2-^{13}C]pyruvate. The derivation of this model is available online (van Heijster FHA, Breukels V, Heerschap A. *Quantitative model of citrate production and secretion in prostate epithelial tissue*, arXiv:1908.11237 [q-bio.MN], June 2021).

The quantitative model takes into account:

- Citrate or citrate equivalents diverging from the Krebs cycle every Krebs cycle turn.
- Anaplerotic inflow of carbons from pyruvate into the Krebs cycle as oxaloacetate.
- Inflow of ^{13}C carbons from pyruvate into the Krebs cycle, via acetyl-CoA.
- The ^{13}C-enrichments of both the pyruvate and acetyl-CoA pool.
- Fast exchange between malate, fumarate and oxaloacetate on one hand, and citrate, isocitrate and α-ketoglutarate on the other hand.
- The dilution of Krebs cycle metabolites by exchange of ^{13}C labeled metabolites with their unlabeled counterparts.

In deriving this model we assumed that:

- Citrate is produced by the condensation reaction of oxaloacetate with acetyl-CoA in the Krebs cycle. ^{13}C carbons of pyruvate can enter the Krebs cycle via the pyruvate carboxylase (PC) route, resulting in labeled oxaloacetate and via the pyruvate dehydrogenase complex (PDC) route, resulting in labeled acetyl-CoA.
- During every cycle turn a fraction d of Krebs cycle metabolites (taken in this model as citrate equivalents) diverges from the cycle, only fraction $(1 - d)$ will continue in the cycle during the next cycle turn. We assume a significant part of this loss is due to citrate secretion, and therefore define this fraction d as the apparent Krebs cycle secretion fraction. If there are no other cataplerotic pathways involved, this fraction d is equal to the true citrate secretion fraction c.
- The ^{13}C-enrichments of both the pyruvate and acetyl-CoA pool are constant over time.
- Anaplerotic inflow of (unlabeled) carbons from e.g. glutamine, aspartate or malate during supplementation with [1,6-$^{13}C_2$]glucose or [2-^{13}C]pyruvate does not contribute to the ^{13}C-labeling pattern of citrate since there is no ^{13}C carbon added to either the ^{13}C pools of pyruvate or acetyl-CoA. Dilution of the Krebs cycle metabolite pools does not have an effect on the labeling but is seen by the model as efflux of citrate equivalents (see assumption below).
- ^{13}C-labeled citrate or citrate equivalents (Krebs cycle metabolites) diverge from the Krebs cycle only to reenter the Krebs cycle through exchange and not at a different position in the Krebs cycle after being converted into another Krebs cycle metabolite (so no short-cuts). Exchange of Krebs cycle metabolites with metabolites outside the Krebs cycle does not change the labeling pattern of the citrate that is secreted, but does lead to loss of citrate equivalents. For example, exchange of oxaloacetate with a potential aspartate pool or the exchange of α-ketoglutarate with the glutamate pool does not change the labeling pattern of the α-ketoglutarate that continues in the Krebs cycle. Even if it continues to be converted to glutamine and back. It does however lead to loss of carbon skeletons (citrate equivalents) if there is net flux of α-ketoglutarate to the glutamate pool. These cataplerotic pathways are thus contributing to the apparent Krebs cycle secretion fraction d and this apparent Krebs cycle secretion fraction is therefore much larger than the real citrate secretion fraction.
- Fast exchange between malate, fumarate and oxaloacetate results in full scrambling of the carbons in oxaloacetate produced via the pyruvate carboxylase pathway.
- Fast exchange between citrate, isocitrate and α-ketoglutarate results in loss of citrate C6 labeling, taken into account by introducing a dilution factor ϵ_c.
- The fraction of carbon skeletons diverging from the Krebs cycle every cycle turn d remains constant over the time period of the experiments.
- If labeled citrate is used for lipid synthesis, this can result in backflow of labels into the Krebs cycle via oxaloacetate, this effect is expected to be negligible and is therefore not taken into account.

Summary of the main features of the model used for the calculation:

- The quantitative model describes the fate of ^{13}C labels after the application of [1,6-$^{13}C_2$]glucose or [2-^{13}C]pyruvate to prostate tissue or epithelial cells.
- The ^{13}C-labeling pattern of the citrate that is secreted is different depending on the number of full Krebs cycle turns that are completed before subsequent secretion into the medium or into the prostatic lumen. The ^{13}C citrate pool in the medium is the sum of all differently ^{13}C-labeled citrate molecules that are secreted in over time. This means that the final ^{13}C distribution over the six carbons in citrate is the sum of all contributions secreted during each different Krebs cycle turn n. The derivation of the expressions describing this distribution are reported in arXiv:1908.11237, and a summary of the results are presented in Table S4. Note that these distributions depend on apparent Krebs cycle secretion fraction d, pyruvate carboxylase fraction f^{PC} (or f^{PDC}, with $f^{PDC} = 1 - f^{PC}$) and dilution factors ϵ_a, ϵ_p and ϵ_c (described below).
- From the ^{13}C NMR spectra obtained from the cell medium after incubation with ^{13}C labeled substrates we calculated two independent experimental ratios R_1 and R_2, described in this manuscript.

$$R_1 = \frac{C_{2/4} - C_3}{C_{2/4}} \qquad [Equation\ S17]$$

$$R_2 = \frac{C_1 + C_5}{C_3} \qquad [Equation\ S18]$$

  These ratios were used in combination with the model in Table 3 (and described in more detail in Van Heijster *et al.*, arXiv:1908.11237), to calculate the apparent Krebs cycle secretion fraction and pyruvate carboxylase fraction.
- The relative carbon labeling at each position of citrate as a function of the apparent secretion fraction d is shown in figure S6A for incubation with [1,6-$^{13}C_2$]glucose and in figure S6B with [2-^{13}C]pyruvate. As described in the manuscript, the ^{13}C-enrichment of the pyruvate pool was found to be 80% after supplementation with either ^{13}C-labeled glucose or pyruvate, and the ^{13}C-enrichment of the acetyl-CoA pool was found to be 56%. Therefore, enrichment factors $\epsilon_p = 0.8$ for pyruvate and $\epsilon_a = 0.56/0.8 = 0.7$ for the acetyl-CoA pool were introduced in the equations to correct for this. An additional dilution factor ϵ_c is seen in the equations for citrate C6, due to partial loss of this carbon during fast exchange between citrate, isocitrate and α-ketoglutarate.

Calculation of ratios, average number of cycles and PC contribution. From the ^{13}C-distribution in citrate after supplementation of cells with [1,6-$^{13}C_2$]glucose we calculate ratio R_1:

$$R_1 = \frac{C_{2/4} - C_3}{C_{2/4}} = \frac{C_2 + C_4 - C_3}{C_2 + C_4} = \frac{\epsilon_a f^{PDC} + \frac{1-d}{1+d}\epsilon_a f^{PDC} + \frac{1}{1+d} f^{PC} - \frac{1-d}{1+d}\epsilon_a f^{PDC} - \frac{1}{1+d} f^{PC}}{\epsilon_a f^{PDC} + \frac{1-d}{1+d}\epsilon_a f^{PDC} + \frac{1}{1+d} f^{PC}} \frac{\epsilon_p}{\epsilon_p} \qquad [Equation\ S19]$$

Which can be rewritten as:

$$\Rightarrow f^{PDC} = \frac{R_1}{\epsilon_a(1+d) - R_1(2\epsilon_a - 1)} \qquad [Equation\ S20]$$

From the ^{13}C-distribution in citrate after supplementation with [2-^{13}C]pyruvate, we can calculate ratio R_2 and subsequently substitute f^{PDC} with the result obtained in Equation 21:

$$R_2 = \frac{C_{1/5}}{C_3} = \frac{C_1 + C_5}{C_3} = \frac{\epsilon_a f^{PDC} + \frac{1-d}{2}\left(\frac{1}{1+d} f^{PC} + \epsilon_a f^{PDC}\right)}{\frac{1}{1+d} f^{PC}} \frac{\epsilon_p}{\epsilon_p}$$

$$= \frac{\frac{R_1}{(1+d) - R_1(2 - \frac{1}{\epsilon_a})} + \frac{1-d}{2}\left(\frac{1}{1+d}\left(1 - \frac{R_1}{(1+d) - R_1(2 - \frac{1}{\epsilon_a})}\right) + \frac{R_1}{(1+d) - R_1(2 - \frac{1}{\epsilon_a})}\right)}{\frac{1}{1+d}\left(1 - \frac{R_1}{(1+d) - R_1(2 - \frac{1}{\epsilon_a})}\right)} \qquad [Equation\ S21]$$

Which we can rewrite to solve for d:

$$-\epsilon_a(R_1 + 1)d^2 + \epsilon_a(4R_1 - 2R_2)d + \epsilon_a(R_1 - 2R_2 + 4R_1R_2 + 1) = 0 \qquad [Equation\ S22]$$

Now we can calculate d using $R_1 = 0.75$, $R_2 = 5.2$ and $\epsilon_a = 0.56/0.8 = 0.7$

$$\begin{aligned} 0 &= -(R_1 + 1)d^2 + (4R_1 - 2R_2)d + (R_1 - 2R_2 + 4R_1R_2 + 1) \\ \Rightarrow 0 &= -1.75d^2 - 7.4d + 6.95 \qquad [Equation\ S23] \\ \Rightarrow d &= 0.7912 \end{aligned}$$

Using the expression for f^{PDC} we can now calculate f^{PC}:

$$f^{PC} = 1 - f^{PDC} = 1 - \frac{R_1}{\epsilon_a(1+d) - R_1(2\epsilon_a - 1)} \Rightarrow f^{PC} = 0.214 \qquad [Equation\ S24]$$

Finally we calculate the average number of turns a citrate molecule makes before leaving the Krebs cycle:

$$< n >= \sum_{n=0}^{n=\infty} n(1-d)^n = \frac{1-d}{d} = 0.264 \qquad [Equation\ S25]$$

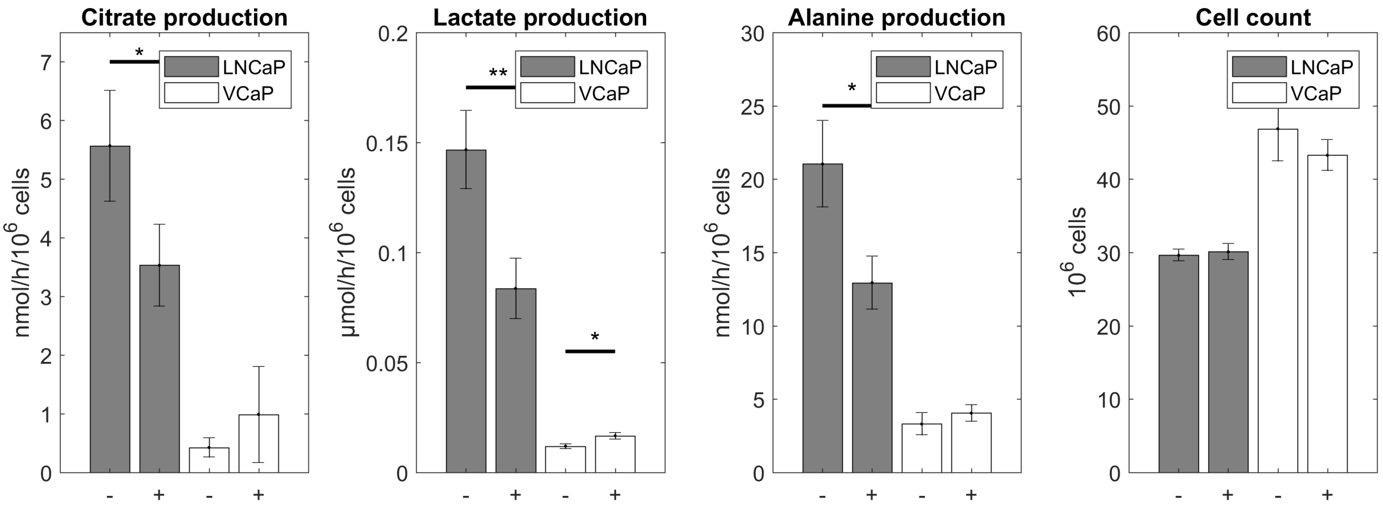

Figure S1. Production rates of citrate, lactate, alanine and cell count for LNCaP and VCaP. Cells grown in RPMI-1640 media supplemented with zinc (50 μM) (N=3), indicated with + and without zinc (N=3), indicated with - (*p<0.05, **p<0.01).

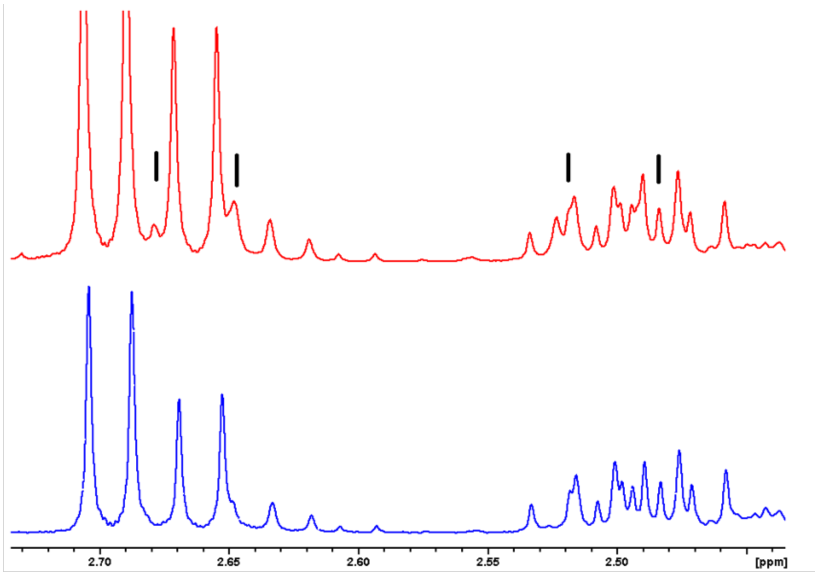

Figure S2 A.) ^{1}H-NMR spectra of RPMI-1640 medium with LNCaP cells (orange) or VCaP cells (blue) grown for 48h after supplementation of the medium with unlabeled aspartate. Signals for citrate are indicated. The experiment is done in parallel with the experiments using [U-$^{13}C_4$]aspartate, to check for production of citrate, since in the labeled experiments the complex J-coupling patterns obscure the citrate peaks.

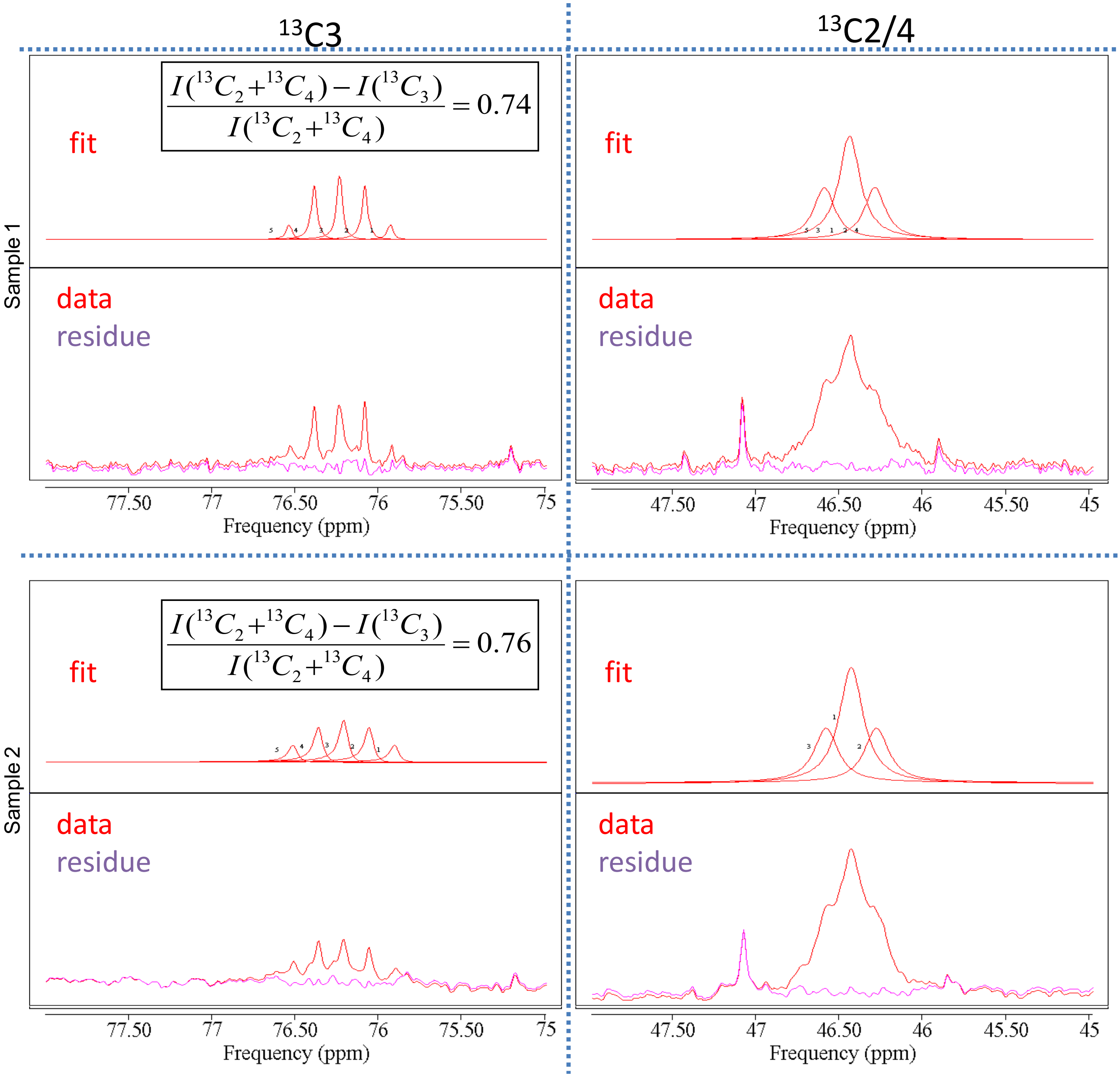

Figure S3. Analysis of the ^{13}C labeling pattern of citrate in growth medium of LNCaP supplemented with [1,6-^{13}C]glucose. Left column: ^{13}C3 region. Right column: ^{13}C2/4 region. Two examples are shown, the experimental ^{13}C-spectra with residue at the bottom and the fitted spectra at the top. A ratio between the integrals of the C2 and C3 signals is calculated, see inset. The ^{13}C-labels of [1,6-^{13}C]glucose end up in citrate at the C2 position via acetyl-CoA. After completing one full round of this cycle it will end up at the C2 or C3 position of oxaloacetate. If then this oxaloacetate undergoes a condensation reaction with another ^{13}C-labled acetyl-CoA to form citrate again, this results in different labeling patterns due to J-coupling between the carbons. Spectra are fitted using *j*MRUI AMARES.

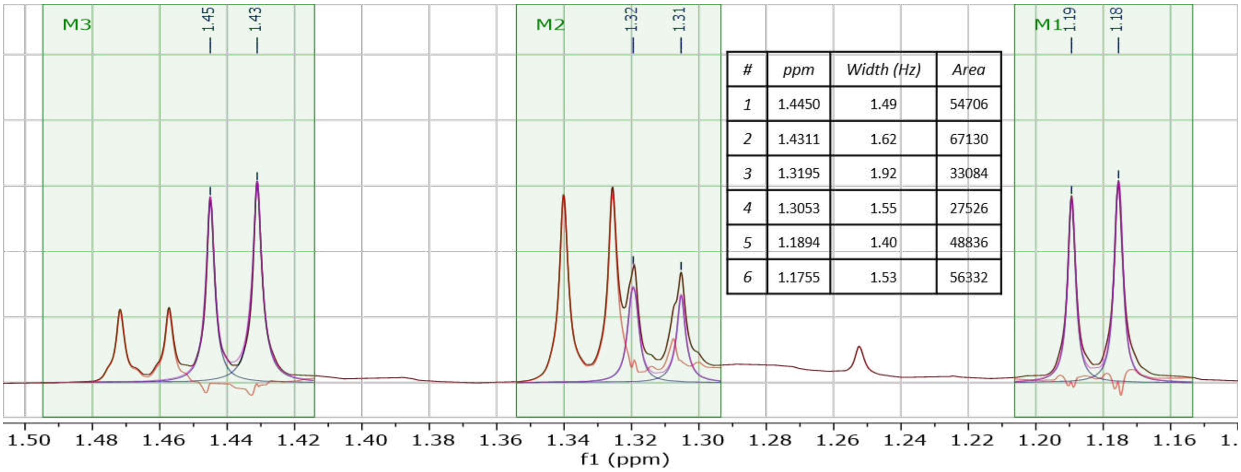

#	ppm	Width (Hz)	Area
1	1.4450	1.49	54706
2	1.4311	1.62	67130
3	1.3195	1.92	33084
4	1.3053	1.55	27526
5	1.1894	1.40	48836
6	1.1755	1.53	56332

Figure S4. ^{1}H-NMR spectrum of lactate in cell extract after supplementation of LNCaP cells with [1,6-$^{13}C_2$]glucose for 48h. The $^{13}C\mathbf{H}_3$ doublets of the methyl group of ^{13}C-labeled lactate at 1.44 ppm and 1.18 ppm, and the $^{12}C\mathbf{H}_3$ doublet of unlabeled lactate at 1.32 ppm are shown and fitted using MestReNova software. From the calculated peak areas it can be calculated that 82% of the lactate in the cells is ^{13}C-labeled.

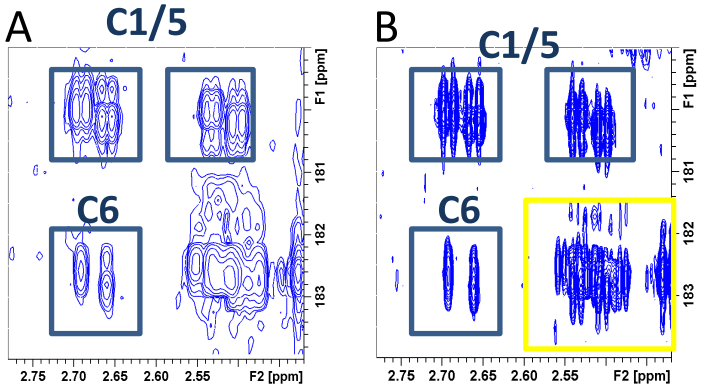

Figure S5. ^{13}C labeling of citrate after supplementation with [5-^{13}C]glutamine. A. ^{1}H-^{13}C-HMBC of LNCaP medium supplemented with [5-^{13}C]glutamine and incubated for 48h. Spiked with citrate (6 mM), the citrate resonances are indicated. In the yellow box right of citrate C6 the overlapping resonances of pyroglutamate are shown that overlap with C6 in figure 8. B. ^{1}H-^{13}C-HMBC of VCaP medium supplemented with [5-^{13}C]glutamine and incubated for 48h and spiked with citrate (6 mM). The citrate resonances are indicated.

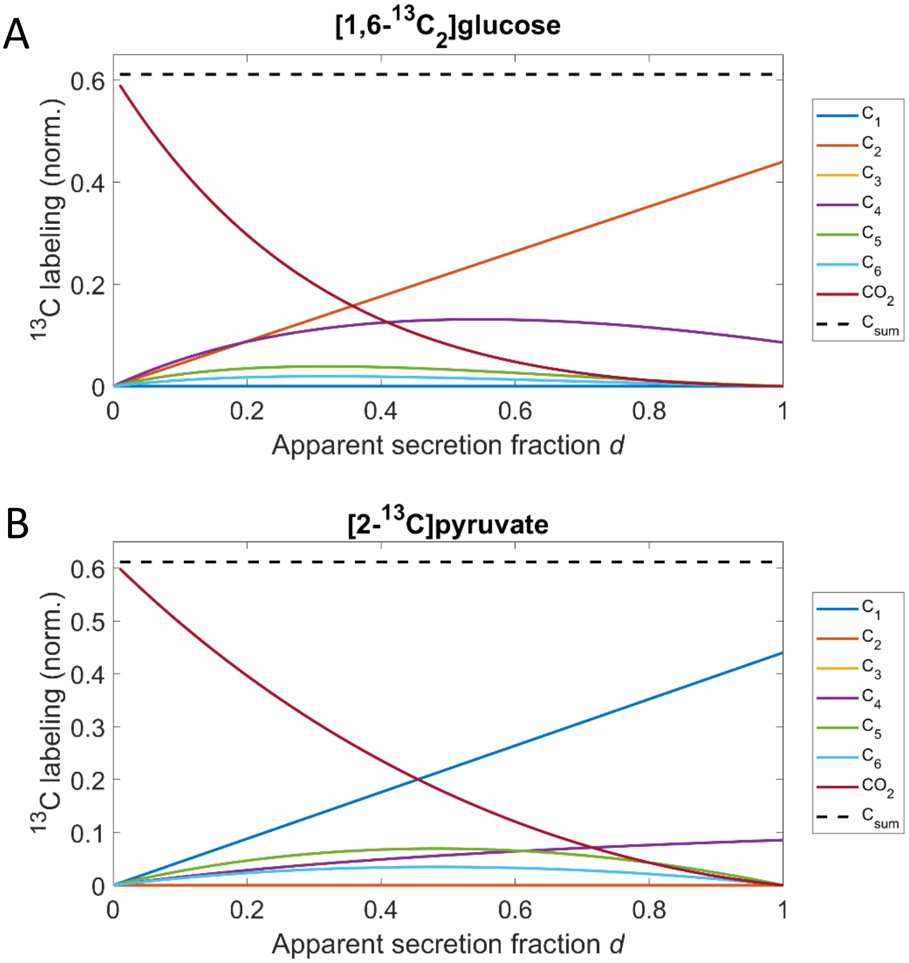

Figure S6. ^{13}C distribution over the six citrate carbons versus apparent secretion fraction d after supplementation of the incubation medium with [1,6-$^{13}C_2$]glucose (A) or [2-^{13}C]pyruvate (B). A pyruvate carboxylase fraction equal to $f^{PC} = 0.21$ together with ^{13}C-enrichments of the pyruvate and acetyl-CoA pools of 0.8 and 0.7 are assumed.

		LNCaP (N=3)	*VCaP (N=3)*
Citrate (nmol/h/10^6 cells)	blanco	5.6 ±1.0	0.4 ±0.2
	+Zn^{2+}	3.5 ±0.7	1.0 ±0.8
Lactate(nmol/h/10^6 cells)	blanco	146.7 ±17.8	11.9 ±1.1
	+Zn^{2+}	83.6 ±13.7	16.6 ±1.5
Alanine(nmol/h/10^6 cells)	blanco	21.0 ±3.0	3.3 ±0.8
	+Zn^{2+}	12.9 ±1.8	4.1 ±0.6
Citrate/ Lactate	Blanco	(3.8 ±0.2)x10^{-2}	(3.5 ±1.2)x10^{-2}
	+Zn^{2+}	(4.2 ±0.5)x10^{-2}	(5.8 ±4.7)x10^{-2}
Alanine / Lactate	Blanco	(14.3 ±0.4)x10^{-2}	(27.7 ±5.0)x10^{-2}
	+Zn^{2+}	(15.5 ±0.4)x10^{-2}	(24.3 ±1.7)x10^{-2}
Citrate / Alanine	Blanco	(26.4 ±0.9)x10^{-2}	(12.6 ±2.1)x10^{-2}
	+Zn^{2+}	(27.2 ±0.3)x10^{-2}	(23.1 ±1.7)x10^{-2}

Table S1. Citrate, lactate and alanine production for both cell lines. Production per million cells, with or without supplementation of 50 µM Zn^{2+} averaged over 48h with standard deviation and metabolite ratios.

Table S2. Relative lactate labeling and glucose consumption of LNCaP and VCaP cells after [1,6-$^{13}C_2$]glucose supplementation.

	$^{12}C/^{13}C$-lactate (^{13}C-glc suppl.)		Acetyl-CoA pool enrichment		Glc consumption (nmol/h/10^6 cells)		^{13}C-lactate/^{13}C-citrate	
	Sample 1	*Sample 2*	*Sample 1*	*Sample 2*	*Sample 1*	*Sample 2*	*Sampl e 1*	*Sample 2*
LNCaP	0.20	0.21	0.56	0.55	241	268	38	70
VCaP	0.26	0.28	0.52	0.49	108	78	68	115

$^{12}C/^{13}C$ ratio in lactate, average glucose consumption of both cell lines at full confluency and ^{13}C-lactate/^{13}C-citrate ratio in ^{13}C-NMR spectrum of growth media after supplementation of the cells with [1,6-$^{13}C_2$]glucose.

Table S3. Ratios of citrate carbons for [2-^{13}C]pyruvate and [5-^{13}C]glutamine experiments and reductive exchange vs. oxidative metabolism fraction for both cell lines.

	**[5-^{13}C]Glutamine experiments**				**[2-^{13}C]pyruvate experiments**
	$C_{1/5}/C_6$	*Krebs cycle vs. red. ex.*	*Reductive exchange fraction*	*Citrate fraction from ^{13}C-Gln (mean ± std. dev.)*	*Ratio $R_2 = C_{1/5}/C_3$ (mean ± std. dev.)*
LNCaP	6.65	1.13-1.41	0.53-0.59	0.26 ±0.09 (N=2)	5.21 ±0.99 (N=5)
VCaP	8.80	1.62-2.03	0.62-0.67	0.29 ±0.06 (N=2)	13.81 ±1.34 (N=2)
Medium + citrate	3.48	n.a.	n.a.	n.a	n.a.

The ratio C1/5/C6 from the ^{1}H-^{13}C-HMBC experiments of the media of LNCaP and VCaP cells supplemented with [5-^{13}C]glutamine and the corresponding ratios for reductive exchange vs. oxidative metabolism are shown (calculated using the C1/5/C6 ratios and equation S11). Next to this, the contribution of (^{13}C-)glutamine to the total citrate production is calculated. The last column shows the ratio R_2 found in the [2-^{13}C]pyruvate experiments. It gives an estimate of pyruvate going via pyruvate dehydrogenase complex (PDH) versus going via pyruvate carboxylase (PC) into the Krebs cycle.

$\overrightarrow{C}(n)$ is the ^{13}C labeling of citrate (and CO_2) at cycle n. The first labeling at $n = 0$ can be summarized for the ^{13}C substrates we use as follows:

1. [1,6-$^{13}C_2$]glucose $\xrightarrow{glycolysis}$ [3-^{13}C]pyruvate $\xrightarrow{PDC}$ [2^{13}C]acetyl-CoA or [1,6-$^{13}C_2$]glucose $\xrightarrow{glycolysis}$ [3-^{13}C]pyruvate $\xrightarrow{PC}$ [2-^{13}C]oxaloacetate/[3-^{13}C]oxaloacetate (due to rapid equilibrium between oxaloacetate, malate and (symmetrical) fumarate). Resulting in citrate labeling at C2 (PDC), C3 or C4 (PC) respectively.

2. [2-^{13}C]pyruvate $\xrightarrow{PDC}$ [1^{13}C]acetyl-CoA or [2-^{13}C]pyruvate $\xrightarrow{PC}$ [2-^{13}C]oxaloacetate/[3-^{13}C]oxaloacetate. Resulting in citrate labeling at C1 (PDC), C3 or C4 (PC) respectively.

$$\overrightarrow{C}(n) = \begin{pmatrix} C_1(n) \\ C_2(n) \\ C_3(n) \\ C_4(n) \\ C_5(n) \\ C_6(n) \\ CO_2(n) \end{pmatrix} d(1-d)^n \qquad [Equation\ S26]$$

$\overrightarrow{^{13}C}_{distr}$	$=$	$\overrightarrow{C}(0)$	$+$	$\overrightarrow{C}(1)$	$+$	$\overrightarrow{C}(2)$	$+...$	$=$	$\sum_{n=0}^{n=\infty} \overrightarrow{C}(n)$
$\overrightarrow{^{13}C}^{glc\text{-}1,6}_{distr}$	$=$	$\begin{pmatrix} 0 \\ \epsilon_a d f^{PDC} \\ \frac{1}{2} f^{PC} \\ \frac{1}{2} f^{PC} \\ 0 \\ 0 \\ 0 \end{pmatrix} \epsilon_p d$	$+$	$\begin{pmatrix} 0 \\ \epsilon_a d f^{PDC} \\ \frac{\epsilon_a d}{2} f^{PDC} + \frac{1}{4} f^{PC} \\ \frac{\epsilon_a d}{2} f^{PDC} + \frac{1}{4} f^{PC} \\ \frac{1}{4} f^{PC} \\ d\epsilon_c \frac{1}{4} f^{PC} \\ 0 \end{pmatrix} \epsilon_p d(1-d)$	$+$	$\begin{pmatrix} 0 \\ \epsilon_a d f^{PDC} \\ \frac{3\epsilon_a d}{4} f^{PDC} + \frac{1}{8} f^{PC} \\ \frac{3\epsilon_a d}{4} f^{PDC} + \frac{1}{8} f^{PC} \\ \frac{\epsilon_a d}{4} f^{PDC} + \frac{1}{8} f^{PC} \\ \frac{\epsilon_a \epsilon_c d}{4} f^{PDC} + \frac{\epsilon_c}{8} f^{PC} \\ \frac{1}{2d} f^{PC} \end{pmatrix} \epsilon_p d(1-d)^2$	$+...$	$=$	$\begin{pmatrix} 0 \\ \epsilon_a f^{PDC} \\ \frac{(1-d)\epsilon_a}{1+d} f^{PDC} + \frac{1}{1+d} f^{PC} \\ \frac{(1-d)\epsilon_a}{1+d} \epsilon_a f^{PDC} + \frac{1}{1+d} f^{PC} \\ \frac{1-d}{2(1+d)} \left((1-d)\epsilon_a f^{PDC} + f^{PC}\right) \\ \frac{(1-d)\epsilon_c}{2(1+d)} \left((1-d)\epsilon_a f^{PDC} + f^{PC}\right) \\ \frac{(1-d)^3 \epsilon_a}{d(1+d)} f^{PDC} + \frac{(1-d)^2}{d(1+d)} f^{PC} \end{pmatrix} \epsilon_p d$
$\overrightarrow{^{13}C}^{pyr\text{-}2}_{distr}$	$=$	$\begin{pmatrix} \epsilon_a d f^{PDC} \\ 0 \\ \frac{1}{2} f^{PC} \\ \frac{1}{2} f^{PC} \\ 0 \\ 0 \\ 0 \end{pmatrix} \epsilon_p d$	$+$	$\begin{pmatrix} \epsilon_a d f^{PDC} \\ 0 \\ \frac{1}{4} f^{PC} \\ \frac{1}{4} f^{PC} \\ \frac{\epsilon_a d}{2} f^{PDC} + \frac{1}{4} f^{PC} \\ \frac{\epsilon_a \epsilon_c d}{2} f^{PDC} + \frac{\epsilon_c}{4} f^{PC} \\ 0 \end{pmatrix} \epsilon_p d(1-d)$	$+$	$\begin{pmatrix} \epsilon_a d f^{PDC} \\ 0 \\ \frac{1}{8} f^{PC} \\ \frac{1}{8} f^{PC} \\ \frac{\epsilon_a d}{2} f^{PDC} + \frac{1}{8} f^{PC} \\ \frac{\epsilon_a \epsilon_c d}{2} f^{PDC} + \frac{\epsilon_c}{8} f^{PC} \\ \epsilon_a f^{PDC} + \frac{1}{2d} f^{PC} \end{pmatrix} \epsilon_p d(1-d)^2$	$+...$	$=$	$\begin{pmatrix} \epsilon_a f^{PDC} \\ 0 \\ \frac{1}{1+d} f^{PC} \\ \frac{1}{1+d} f^{PC} \\ \frac{1-d}{2} \left(\frac{1}{1+d} f^{PC} + \epsilon_a f^{PDC}\right) \\ \frac{(1-d)\epsilon_c}{2} \left(\frac{1}{1+d} f^{PC} + \epsilon_a f^{PDC}\right) \\ \frac{(1-d)^2 \epsilon_a}{d} f^{PDC} + \frac{(1-d)^2}{d(1+d)} f^{PC} \end{pmatrix} \epsilon_p d$

Table S4.
^{13}C distributions over six citrate carbons and carbon dioxide for $n = 0 - 2$ and the result of summation of contributions of all Krebs cycle turns. Top row is after supplementation of incubation medium with [1,6-$^{13}C_2$]glucose and bottom row shows the result after supplementation with [2-^{13}C]pyruvate. With f^{PDC} the fraction of pyruvate entering the Krebs cycle via the PDC route, f^{PC} via the PC route, d the apparent Krebs cycle secretion fraction, ϵ_p the ^{13}C-enrichment of the pyruvate pool, ϵ_a of the acetyl-CoA pool, and ϵ_c the loss of C6 carbon as CO_2 due to fast exchange in α-KG-isocitr.-citr. pool

Table S5. Relative signal integrals of metabolites present in medium after [1,6-$^{13}C_2$]glucose supplementation

Metabolite	Lac		Ala	Pro	Gly	Cit		Ac	Glu	Glc	Glyc
Carbon	C2	C3	C3	C4	C2	C3	C2+C4	C2	C4	C6	C1+C3
Integral	6.0	158.8	22.5	1.8	0.8	1.0	1.0	6.3	3.4	11.7	32.3

Relative ^{1}H-^{13}C HMBC integrals of metabolites shown in Fig. 4, present in RPMI-1640 incubation medium after supplementation of LNCaP with [1,6-$^{13}C_2$]glucose for 48h.
